# A novel miR-375-HOXB3-CDCA3/DNMT3B regulatory circuitry contributes to leukemogenesis in acute myeloid leukemia

**DOI:** 10.1186/s12885-018-4097-z

**Published:** 2018-02-13

**Authors:** Laixi Bi, Bin Zhou, Haiying Li, Licai He, Chunjing Wang, Zhonggai Wang, Liqing Zhu, Mengqian Chen, Shenmeng Gao

**Affiliations:** 1 Department of Hematology, The First Affiliated Hospital of Wenzhou Medical University, Nanbaixiang, Ouhai District, Wenzhou, Zhejiang Province 325000 China; 2 Laboratory of Internal Medicine, The First Affiliated Hospital of Wenzhou Medical University, Shangcai Village, Nanbaixiang, Ouhai District, Wenzhou, Zhejiang Province 325000 China; 3 School of Laboratory Medicine & School of Life Science, Wenzhou Medical University, Nanbaixiang, Ouhai District, Wenzhou, Zhejiang Province China; 4 Department of Clinical Laboratory, The First Affiliated Hospital of Wenzhou Medical University, Nanbaixiang, Ouhai District, Wenzhou, Zhejiang Province 325000 China; 5 Department of Drug Discovery and Biomedical Sciences, University of South Carolina College of Pharmacy, Columbia, SC USA

**Keywords:** miR-375, HOXB3, DNMT3B, DNA hypermethylation, AML

## Abstract

**Background:**

Acute myeloid leukemia (AML) is a heterogeneous group of hematopoietic malignancies due to sophisticated genetic mutations and epigenetic dysregulation. MicroRNAs (miRNAs), a class of small non-coding RNAs, are important regulators of gene expression in all biological processes, including leukemogenesis. Recently, miR-375 has been reported to be a suppressive miRNA in multiple types of cancers, but its underlying anti-leukemia activity in AML is largely unknown.

**Methods:**

Quantitative reverse transcriptase PCR (qRT-PCR) was used to measure the expression of miR-375 and *HOXB3* in leukemic cells and normal controls. Targets of miR-375 were confirmed by western blot and luciferase assay. Phenotypic effects of miR-375 overexpression and HOXB3 knockdown were assessed using viability (trypan blue exclusion assay), colony formation/replating, as well as tumor xenograft assays in vivo.

**Results:**

The expression of miR-375 was substantially decreased in leukemic cell lines and primary AML blasts compared with normal controls, because DNA hypermethylation of precursor-miR-375 (pre-miR-375) promoter was discovered in leukemic cells but not in normal controls. Lower expression of miR-375 predicted poor outcome in AML patients. Furthermore, forced expression of miR-375 not only decreased proliferation and colony formation in leukemic cells but also reduced xenograft tumor size and prolonged the survival time in a leukemia xenograft mouse model. Mechanistically, overexpression of miR-375 reduced HOXB3 expression and repressed the activity of a luciferase reporter through binding 3′-untranslated regions (3’-UTR) of *HOXB3* mRNA. Overexpression of HOXB3 partially blocked miR-375-induced arrest of proliferation and reduction of colony number, suggesting that HOXB3 plays an important role in miR-375-induced anti-leukemia activity. Knockdown of *HOXB3* by short hairpin RNAs reduced the expression of cell division cycle associated 3 (CDCA3), which decreased cell proliferation. Furthermore, HOXB3 induced DNA methyltransferase 3B (DNMT3B) expression to bind in the pre-miR-375 promoter and enhanced DNA hypermethylation of pre-miR-375, leading to the lower expression of miR-375.

**Conclusions:**

Collectively, we have identified a miR-375-HOXB3-CDCA3/DNMT3B regulatory circuitry which contributes to leukemogenesis and suggests a therapeutic strategy of restoring miR-375 expression in AML.

**Electronic supplementary material:**

The online version of this article (10.1186/s12885-018-4097-z) contains supplementary material, which is available to authorized users.

## Background

Acute myeloid leukemia (AML) is characterized by the blockage of differentiation and uncontrolled proliferation due to various genetic mutations and epigenetic dysregulation [1]. Although current therapy involves intensive chemotherapy and stem cell transplantation, AML is still fatal in about half of younger patients and approximately 80% of patients over the age of 60 because of primary refractoriness, relapse, or treatment-related mortality [2]. Chromosome translocations leading to fusion genes such as *MLL-AF9*, *PML-RARα*, and *AML1-ETO* as well as various genetic mutations such as *FLT3*, *Kit*, *TET2*, and *IDH1* contribute to the pathogenesis of AML [3]. However, recently emerging discoveries have indicated that epigenetic dysregulations including DNA hypermethylation and non-coding RNAs such as miRNAs play an important role in the pathogenesis of AML [4].

MicroRNAs (miRNAs) are a class of noncoding RNAs with 21 nucleotides. MiRNAs directly bind 3′-untranslational region (UTR) of messenger RNAs (mRNAs) of target genes, resulting in translational repression or mRNA degradation [5]. MiRNAs have recently been found to play an important role in the biological regulations such as apoptosis, proliferation, and differentiation in hematological cells by modulating the expression of oncogenes or tumor suppressors [6]. Dysregulation of miRNAs is involved in the pathogenesis of leukemia and miRNAs have rapidly emerged as novel therapeutic targets [7]. For example, decreased expression of miR-193a facilitates the leukemogenesis through activating PTEN/PI3K signaling pathway [8]. Most studies demonstrate that miR-375 acts as tumor suppressor gene and is downregulated in various types of cancers, including oral squamous cell carcinoma [9], gastric cancer [10], and colorectal cancer [11]. However, miR-375 is upregulated in prostate cancer and miR-375 acts as oncogene to enhance tumor progression [12]. Our published data demonstrate that miR-375 is decreased in patients with myeloproliferative neoplasm (MPN) compared with normal controls. Overexpression of miR-375 suppresses cell proliferation and decreases colony formation in hematopoietic progenitors from MPN patients [13]. These results demonstrate that miR-375 acts as either a tumor suppressor or an oncogene in different contexts. However, the potential role of miR-375 in leukemia is largely unknown.

The homeobox (*HOX*) genes, which are mainly involved in development, are highly conserved subgroup of the homeodomain transcription factors. *HOX* genes are divided into four different families (*HOXA*, *B*, *C*, and *D*) according to its different position in four chromosomes [14]. Aberrant expression of *HOX* has been reported in abnormal development and malignancy. For example, increased expressions of *HOXB3*, *B4*, and *A7–11* are found in the most primitive progenitors of AML [15]. *HOXB4* expression is elevated in a group of AML patients and higher *HOXB4* expression is associated with better outcome [16]. The mRNA and protein expressions of HOXB3 are significantly increased in primary prostate cancer tissues compared with the adjacent normal prostate tissues. Furthermore, overexpression of HOXB3 promotes prostate cancer proliferation through transcriptional activation of cell division cycle associated 3 (*CDCA3*) [17]. As a transcript factor, HOXB3 contributes to cancer cell proliferation and metastasis through activating DNA methyltransferase (*DNMT3B*) and subsequent inactivation of the *RASSF1A* tumor suppressor gene [18]. However, the biological role of HOXB3 in AML is still largely unclear.

Here, we report a new miR-375-HOXB3-CDCA3/DNMT3B regulatory circuitry in leukemic cells. DNA hypermethylation of pre-miR-375 promoter results in the low expression of miR-375, which enhances proliferation of leukemic cells through upregulation of HOXB3. The increased expression of HOXB3 recruits DNMT3B to further facilitate DNA hypermethylation of the pre-miR-375 promoter, leading to the lower expression of miR-375. Thus, restoring the expression of miR-375 blocks HOXB3-CDCA3/DNMT3B regulatory circuitry and finally inhibits cell growth and colony formation in leukemic cells.

## Methods

### Leukemic cell lines, primary AML blasts, umbilical cord blood (UCB), and reagents

Human leukemic cell lines including THP1 and HL-60 (ATCC, Manassas, VA, USA) were purchased for the present study. Leukemic cell lines were cultured in RPMI 1640 supplemented with 10% fetal bovine serum (Invitrogen, Carlsbad, CA, USA) and 1% penicillin-streptomycin in humidified 37 °C incubator with 5% CO_2_. Bone marrow mononuclear cells from AML patients and mononuclear cells from umbilical cord blood (UCB) were isolated by Ficoll density gradient centrifugation (GE Healthcare, Uppsala, Sweden) and cultured in StemSpan SFEM (StemCell Technologies, Vancouver, Canada), which was supplemented with human SCF, Flt3 ligand, erythropoietin, IL-3, and IL-6 at final concentrations of 10 ng/ml. UCB samples were received by the normal donor repository in the Laboratory of Internal Medicine at the First Affiliated Hospital of Wenzhou Medical University. Normal CD34^+^ cells from normal controls (NC) were isolated and enriched by immunomagnetic positive selection kit (Stem Cell Technologies, Vancouver, Canada). Cell viability was determined by the trypan-blue exclusion assay. All procedures involving human participants were in accordance with the ethical standards of the Ethics Committee of the First Affiliated Hospital of Wenzhou Medical University and the Declaration of Helsinki. All the patients gave informed consent. 5′-azacytidine (AZA, Sigma-Aldrich, St Louis, MO, USA) was dissolved in distilled water and kept at − 20 °C until used.

### Cohort of AML patients

A total of 102 patients (Additional file 1: Table S1, Age ≤ 60) between October 2012 and October 2015 with de novo AML at the First Affiliated Hospital of Wenzhou Medical University were included. Patients were diagnosed and classified according to the French–American–British (FAB) criteria. This study was approved by the institutional ethics committee and a written informed consent in accordance with the recommendations of the Declaration of Helsinki. All AML patients (excluding M3 subtype) received first-line treatment with standard induction chemotherapy, which consisted of daunorubicin 60–90 mg/m^2^ or idarubicin 12 mg/m^2^ on days 1–3 and cytarabine 100–200 mg/m^2^ on days 1–7. One or two courses of induction chemotherapy were given to patients to get complete remission (CR). AML patients were treated with 3–4 cycles of high-dose cytarabine in the consolidation therapy. For the patients who were refractory or relapsed, salvage therapy was taken. Cytogenetic analyses of the samples were performed at diagnosis through G-banding. The criteria used to describe a cytogenetic clone and description of karyotype followed the recommendations of the International System for Human Cytogenetic Nomenclature [19]. Fusion genes such as *AML1-ETO* were measured by qRT-PCR [20] and gene mutations such as *WT1* and *c-Kit* were performed on all of the samples by direct sequencing as previously reported [3]. Bone marrow mononuclear cells from these 102 AML patients were isolated and the expression of miR-375 was detected. The median expression of miR-375 was used as the cutoff. Survival probabilities were estimated by the Kaplan-Meier method and differences in survival distributions were compared using the log-rank test. Overall survival (OS) was defined as being from the date of diagnosis to death or last follow-up, and disease-free survival (DFS) was defined as the time in complete remission (CR) to relapse or death due to progressive disease.

### RNA extraction and quantitative RT-PCR

Total RNA was extracted by the miRNeasy extraction kit (Qiagen, Valencia, CA, USA) and was used as a template to synthesize cDNA for quantitative RT-PCR (qRT-PCR) analysis in a 7500 real-time PCR system (Applied Biosystems, Carlsbad, CA, USA). Mature miR-375, miR-126, and U6 snRNA were reversely transcribed using Stem-loop RT primer with miScriptIIRT Kit (Qiagen, Valencia, CA, USA). Pre-miR-375 was measured by miScript Precursor Assays (Qiagen). qPCR with SYBR Green dye (Qiagen) was used to determine the expression of mRNA and miRNA. GAPDH and U6 snRNA were used as endogenous controls for qRT-PCR of mRNA and miRNA, respectively. The relative expression levels were evaluated using the 2^-ΔΔCt^ method. Each sample was run in triplicate. All the primer sequences were shown in Additional file 2: Table S2.

### Western blot

Western blot analysis was performed using standard techniques. Briefly, transiently transfected leukemia cells were harvested and lysed by RIPA buffer (Thermo Scientific, Waltham, MA, USA). Proteins (40 μg) from the lysate were fractionated by electrophoresis through polyacrylamide gels (Bio-Rad, Richmond, CA, USA) and transferred to PVDF membranes. Blots were incubated with primary antibodies over night at 4 °C and incubated with second HRP-conjugated antibody for 1 h. Signals were measured by chemiluminescence reagents (Bio-Rad) with imaging system (Bio-Rad). The following antibody was used: HOXB3 (ab82945, Abcam, Cambridge, MA, USA); CDCA3 (ab166902, Abcam); DNMT3B (ab176166, Abcam). As necessary, blots were stripped and reprobed with β-actin antibody (Santa Cruz Biotechnology, Santa Cruz, CA, US) as an endogenous control.

### Cell cycle analysis

About 2×10^6^ leukemic cells were washed and resuspended in PBS solution containing 0.04 mg/ml propidium iodide (Invitrogen) and 100 mg/ml RNase. The samples were analyzed by flow cytometry (Becton Dickinson, Mountain View, CA) using the CELLQuest program (Becton Dickinson) after incubation at room temperature for 30 min. Modifit software (Verity Software House, Topsham, ME, USA) was used to generate histogram to determine number of cells in each phase of the cell cycle.

### Luciferase reporter and mutagenesis assays

293 T cells were seeded in 24-well plates at a density of 1.0×10^5^/ml per well, followed by growth for 24 h before transfection. Each well was transiently cotransfected with 100 ng pMIR-REPORT plasmid bearing the wide-type 3’UTR of *HOXB3* or the 3’UTR with mutated miR-375 binding site of *HOXB3*, 60 pmol scramble (negative control), or miR-375 mimics (GenePharma, Shanghai, China) as well as 10 ng endogenous control vector pRL-SV40 (Promega, Madison, WI, USA) using Hiperfect transfection reagent (Qiagen). Firefly and Renilla luciferase activities were measured by the Dual-Luciferase Reporter Assay System (Promega) in cell lysates harvested after transfection for 24 h. The value of relative luciferase activity indicates the Firefly luciferase activity normalized to that of Renilla luciferase for each assay. Each experiment was performed in triplicate and repeated three times.

### Methylation analysis

Genomic DNA from leukemic cells was extracted using DNA Purification Kit (QIAGEN). For the treatment with sodium bisulfite, DNA (1 mg) was modified by EpiTect Bisulfite Kit (QIAGEN). For bisulfite-sequencing analysis, upstream flanking sequence from − 260 bp − + 136 bp in the pre-miR-375 gene was amplified by PCR using the sodium bisulfite-treated DNA as template. PCR products were subcloned into pUC18 vector and five constructed pUC18 vectors were used to sequence for each sample. The methylation frequencies were calculated as methylated C/ (methylated C + unmethylated C) in every clone. For methylation-specific PCR analysis, the methylation-specific and unmethylation-specific primers were designed by MethPrimer software [21]. Primer sequences are shown in Additional file 2: Table S2.

### Inhibition of miR-375

Before transfection, leukemic cells were seeded in 6-well plates at a density of 2.0 × 10^5^/ml per well. 100 pmol special miR-375 inhibitor Agomir (GenePharma, Shanghai, China) or negative control was transiently transfected into leukemic cell lines by Hiperfect transfection reagent (Qiagen). After transfection for 48 h, cells were collected and proteins were extracted for western blot.

### Construction of plasmids

The paired primers based on the primary sequence of hsa-miR-375 and its flanking regions were cloned into murine stem cell virus vector pMSCV-puro (Clontech, Palo Alto, CA, USA) to construct the plasmid that expresses miR-375. To produce plasmids expressing HOXB3 and DNMT3B, human *HOXB3* and *DNMT3B* coding sequences were amplified by PCR and then cloned into lentivirus vector pLVX-IRES-ZsGreen1 (Clontech) and pMSCV-puro (Clontech), respectively. To construct pMIR-HOXB3 3′UTR plasmid, human *HOXB3* 3′UTR was amplified by PCR and cloned into pMIR-REPORT vector (Ambion, Dallas, TX, USA). The mutation on miR-375-binding sites in human *HOXB3* 3′UTR was generated by the site-directed mutagenesis kit (Stratagene). All the primer sequences were shown in Additional file 2: Table S2. All of these plasmids were confirmed by sequencing.

### Retrovirus production and cell transduction

HEK293 T cells (2×10^6^) were plated in 10 cm dish. After 24 h, all constructed plasmids together with negative control vectors were co-transfected with packaging plasmids into HEK293 T cells. Virus was harvested from the supernatant at 48 h after transfection and further filtered through a 0.45 μm low protein-binding-polysulfonic filter (Millipore, Billerica, MA, USA). Leukemic cells (2×10^5^/ml) were suspended in viral supernatant with 8 μg/ml polybrene (Sigma-Aldrich, St. Louis, MO, USA) and centrifuged at 2000 rpm for 2 h. Puromycin (2 μg/ml, Medchemexpress, Princeton, NJ, USA) was added into the supernatant for 1 week to select positive clones.

### RNA interference

Gene-specific short hairpin RNAs (shRNAs) for *HOXB3*, *DNMT3B*, and *CDCA3* were designed and cloned into pSIREN-RetroQ (Clontech) retroviral vectors. Control shRNA is a nonfunctional construct provided from Clontech. The sequences of shRNAs were shown in Additional file 2: Table S2. These vectors were co-transfected with packaging plasmids (Gap-pol and VSV-G) into 293 T cells to produce retrovirus. Supernatants containing retrovirus were collected at 48 h after transfection and were utilized to transduce leukemic cells.

### Colony formation assay

Bone marrow mononuclear cells (blasts% > 70%) from six AML patients were harvested. CD34^+^ cells were enriched by immunomagnetic positive selection kit (Stem Cell Technologies), followed by the retrovirus transduction of MSCV-miR-375 or MSCV-NC. Retrovirus-transduced CD34^+^ cells (5×10^3^) were seeded on a 35-mm dish with 1.0 ml of methylcellulose medium (MethoCult™ H4434 Classic, Stem Cell Technologies). HL-60 and THP1 cells, which were transduced with MSCV-miR-375 or MSCV-NC, were seeded in the same methylcellulose medium (Stem Cell Technologies). Normal CD34^+^ cells were isolated and enriched from UCB, followed by the retrovirus transduction of MSCV-miR-375 or MSCV-NC and then were plated in the same methylcellulose medium (Stem Cell Technologies). Colonies were scored 10–14 days after plating according to the manufacturer’s protocol.

### Colony-forming/replating assays in vitro

Primitive hematopoietic progenitor cells were harvested and enriched by the Mouse Lineage Cell Depletion Kit (Stem Cell Technologies) from a cohort of 6-week-old C57 mice on the sixth day after 5-fluorouracil treatment. An aliquot of enriched hematopoietic progenitor cells was added to retroviral supernatant together with polybrene in six-well-plate, which were centrifuged at 2000×g for 2 h. The media was replaced with fresh media and incubated for 24 h at 37 °C. Next day, the same procedure was repeated once. Then, an equivalent of 2.0 × 10^3^ cells was plated onto a 35-mm dish with 1.0 ml of methylcellulose medium (MethoCult™ GF M3434, Stem Cell Technologies). The colonies were replated every 7 days under the same condition. The colony-forming/replating assays were repeated three times.

### Chromatin immunoprecipitation assay

The binding activity of DNMT3B in pre-miR-375 promoter was examined by Chromatin Immunoprecipitation (ChIP) Assay Kit (Merck-Millipore, Billerica, MA, USA) according to the manufacturer’s instruction. Briefly, treated and untreated leukemic cells were cross-linked with 1% formaldehyde for 10 min. Chromatin from nuclear extracts was sonicated to generate 200–1000 bp DNA fragments. DNA-protein complexes were immunoprecipitated with 5 μg of specific anti-DNMT3B (ab176166, Abcam). DNA was purified after the DNA-protein cross-link was reversed by heating at 65 °C for 4 h. Standard PCR reactions were performed by primer pairs (Additional file 2: Table S2). The immunoprecipitated DNA was analyzed by qRT-PCR and the amount of precipitated DNA was calculated as the percentage of the input sample.

### Tumorigenicity in nude mice

Male athymic nude mice were purchased from SLAC (Shanghai SLAC Laboratory Animal, Shanghai, China). The animals were maintained under specific pathogen-free (SPF) conditions in the Animal Facility of Wenzhou Medical University. Animal-related experiments were performed according to the Guide for the Care and Use of Laboratory Animals (National Institutes of Health Publications) and approved by the committee for human treatment of animals at Wenzhou Medical University Institutional Guidelines. Six-week-old mice (*N* = 12) were randomly divided into two equal groups. A total of 1×10^7^ viable HL-60-MSCV-NC or HL-60-MSCV-miR-375 cells were resuspended with 200 μl sterile 1× PBS and injected subcutaneously into the right flank of each nude mouse. Both the long and short diameters of xenografts were measured using vernier calipers every 3 days after 2 weeks. When the experiments were terminated at 6 weeks after tumor cell inoculation, all mice were sacrificed and the tumors from each mouse were harvested and weighed. Tumor volumes were measured using the eq. V (mm^3^) = AхB^2^/2, where A is the largest diameter and B is the perpendicular diameter. Tumor lysates were prepared for western blot.

### Engraftment of NOD/SCID-IL2Rγ mice (NSG)

THP1 cells were transduced with lentivirus vector pLVX-IRES-ZsGreen1 (Clontech), followed by sorting to obtain GFP-positive cells. GFP-positive THP1 cells were transduced with MSCV-miR-375 or MSCV-NC, followed by puromycin selection for 1 week. Busulfan (30 mg/kg; B2635; Sigma) was intraperitoneally given to eight-week-old NSG mice 1 day before xenotransplantation. 4×10^5^ THP1-GFP-miR-375 cells or THP1-GFP-NC cells were intravenously injected into NSG mice (*N* = 10). When the mice became moribund, they were humanly sacrificed and survival was evaluated from the first day of the experiment until death.

### Statistical analysis

Data are presented as mean ± SD. Generally, a two-tailed Student’s t-test was employed to evaluate the differences between groups. Differences are considered as significant when the *P* value is < 0.05. All statistical analyses were performed by SPSS 22.0 software (SPSS Inc., Chicago, IL, USA).

## Result

### miR-375 is downregulated in leukemia

**Fig. 1:**
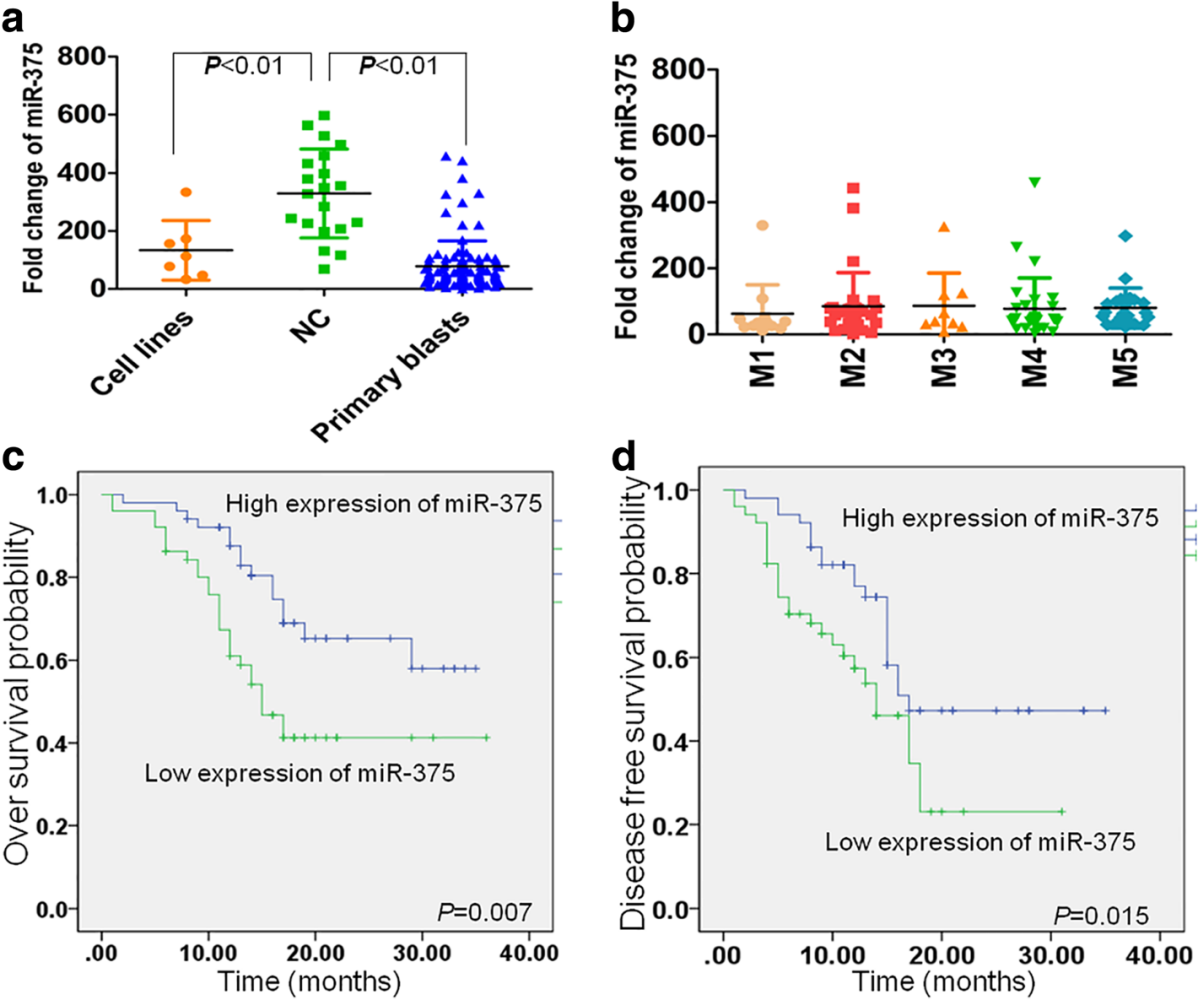
Decreased expression of miR-375 in AML patients predicts poor clinical outcome. **a** MiR-375 expressions were detected by qRT-PCR in several leukemic cell lines including NB4, HL-60, Kasumi-1, HEL, THP1, U937, and K562, 102 primary blasts from AML patients, and 20 normal controls (CD34^+^ cells, NC) by qRT-PCR. The lowest expression of miR-375 in one AML blast was set to 1.0 and then all other specimens were normalized by this lowest specimen. Housekeeping gene U6 is used as a reference. **b** MiR-375 expressions were detected in primary AML blasts according to FAB subtype (M1–M5). (**c** and **d**) The median expression of miR-375 was used as the cutoff. Kaplan-Meier overall survival curve (**c**) and disease-free survival curve (**d**) were indicated according to miR-375 expression level

To determine whether miR-375 is decreased in leukemic cells as reported in solid tumors [10, 22], we measured the levels of miR-375 in 7 leukemic cell lines, 102 primary AML blasts, and 20 CD34^+^ hematopoietic stem and progenitor cells (HSPC) from normal controls (NC). MiR-375 expression was about 60% lower in leukemic cell lines than in NC (*P* < 0.01) and about 76% lower in primary AML blasts than in NC (*P* < 0.01, Fig. 1a), respectively. We further analyzed the expression of miR-375 according to FAB subtype. No significant differences of miR-375 were found between different FAB subtypes from M1–M5 (Fig. 1b). To confirm that miR-375 is virtually expressed in HSPC, the level of a well-described stem cell gene miR-126 [23] was measured in 20 CD34^+^ cells from NC. The average expression of miR-126 is 1.34-fold higher than miR-375 in CD34^+^ cells (Additional file 3: Figure S1A, *P* = 0.0696). Because miR-126 is well expressed in HSPC and no difference was observed between miR-375 and miR-126 expressions, we speculate that miR-375 is virtually expressed in HSPC.

The low expression of miR-375 in AML cells prompted us to determine whether miR-375 expression is associated with clinical outcome in our adult AML cohort. AML patients were divided into two different groups according to the median miR-375 level. MiR-375 expression above or below the median was considered as higher or lower expression. As shown in Fig. 1c and d, AML patients with higher expression of miR-375 predicted better overall survival (OS, HR = 2.311; 95% CI =1.226–4.357; *P* = 0.007) and disease-free survival (DFS, HR = 1.997; 95% CI = 1.115–3.577; *P* = 0.015) compared with those with lower miR-375 expression, respectively. Furthermore, multivariate analysis indicated that the prognostic impact of miR-375 expression on OS and DFS was independent of age, sex, karyotype, and gene mutations including *FLT3, CEBPA,* and *NPM* (Additional file 4: Table S3).

### DNA hypermethylation leads to low expression of miR-375 in leukemic cells

**Fig. 2:**
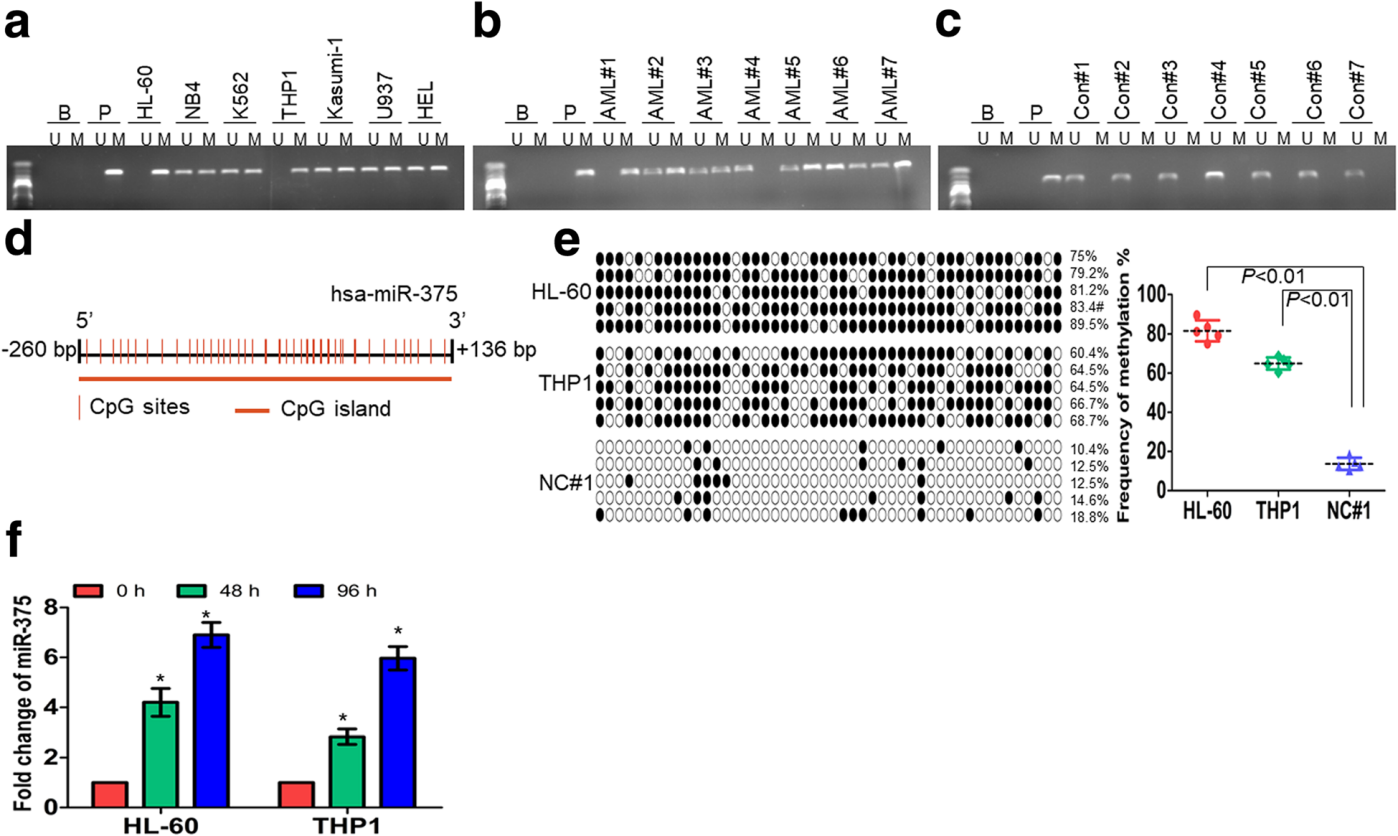
DNA hypermethylation results in the low expression of miR-375. **a**–**c** The methylation status of miR-375 was analyzed by MSP in 7 leukemic cell lines (**a**), 40 primary AML blasts (**b**), and 20 normal controls (**c**). B: Blank; P: positive control of methylated DNA. Bands of ‘M’ or ‘U’ are PCR products amplified by methylation-specific or unmethylation-specific primers, respectively. Shown are the representative figures for primary AML blasts and normal controls. **d** A CpG map of the sequenced region was analyzed by MethPrimer software. **e** Bisulfite genomic sequencing was performed to detect the methylation status of the DNA sequences at − 260 bp − + 136 bp in the pre-miR-375 gene upstream region in HL-60, THP1, and one normal control (NC#1). Five PCR products were shown for each sample. Each row of circles represents the sequence of an individual clone. Black circles and empty circles represent methylated and unmethylated CpG dinucleotides, respectively (Left). Shown was the summary of frequencies of methylated CpG dinucleotides detected in HL-60, THP1, and one NC by bisulfite genomic sequencing (Right). **f** The expression of miR-375 was detected in HL-60 and THP1 cells treated with 5 μM AZA for 2 and 4 days. *P < 0.01 versus untreated cells

Recently, emerging data have indicated that gene silencing is frequently caused by promoter hypermethylation in multiple types of cancers [24, 25]. Several typical CpG islands were found around the region encoding pre-miR-375 by MethPrimer software [21], suggesting that low expression of miR-375 might be due to DNA hypermethylation (Additional file 3: Figure S1B). To determine whether CpG islands of pre-miR-375 were hypermethylated in a tumor-specific manner, the methylation status of miR-375 was analyzed by methylation-specific PCR (MSP) in 7 leukemic cell lines, 40 primary AML blasts, and 20 NC (CD34^+^). Hypermethylation of miR-375 was observed in all 7 leukemic cell lines (Fig. 2a) and in 35 of 40 (87.5%) primary AML blasts (Fig. 2b). In contrast, a complete absence of miR-375 methylation was found in 20 NC (Fig. 2c). We further confirm that these CpG islands were densely methylated by bisulfite sequencing. A CpG map of the sequenced region comprising -260 bp − + 136 bp in the pre-miR-375 gene was indicated in Fig. 2d. CpG islands were densely methylated in HL-60 and THP1 cells but not in one NC (NC#1) by bisulfite sequencing (Fig. 2e, Left). The frequencies of miR-375 methylation in HL-60 and THP1 cells were about 82% and 65%, respectively, compared with 14% in NC#1 (Fig. 2e, Right). To explore whether DNA hypermethylation leads to the silencing of miR-375, the expression of miR-375 was measured in HL-60 and THP1 cells treated with DNA methyltransferase inhibitor 5′-azacytidine (AZA). AZA increased the expression of miR-375 in HL-60 and THP1 cells (Fig. 2f).

### miR-375 targets HOXB3

**Fig. 3:**
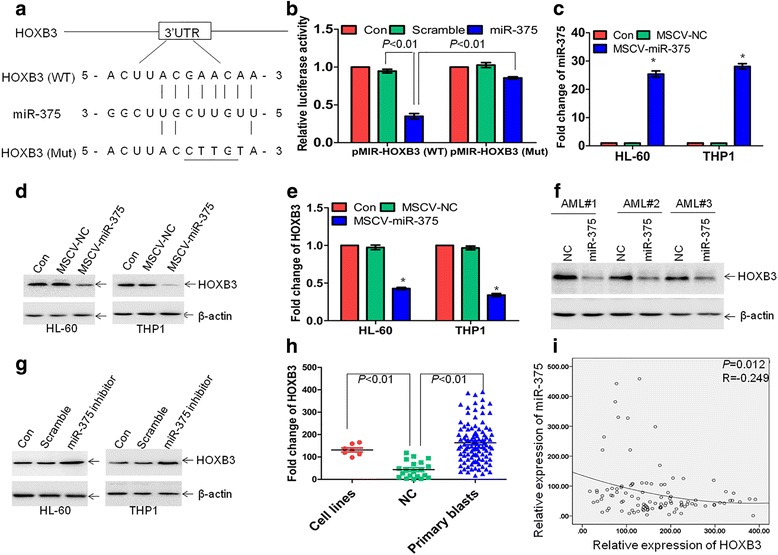
MiR-375 targets HOXB3 via binding 3′-UTR of *HOXB3*. **a** Schematic of putative binding sites for miR-375 in 3′-UTR of *HOXB3*. **b** 293 T cells were transfected with wide-type pMIR-HOXB3UTR (WT), pMIR-HOXB3UTR (Mut), pMIR-NC, and pRL-SV40 containing Renilla luciferase gene for 24 h, followed by the transfection with miR-375 mimic or Scramble for another 24 h. Firefly and Renilla luciferase activities were both detected and histograms showed that the Firefly luciferase activities were normalized to Renilla luciferase activities. **c** The expression of miR-375 was detected in HL-60 and THP1 cells transduced with MSCV-miR-375 or MSCV-NC. *P < 0.01 versus MSCV-NC. (**d** and **e**) HOXB3 protein and mRNA expressions were detected by western blot and qRT-PCR in HL-60 and THP1 cells following transduction with MSCV-miR-375 or MSCV-NC, respectively. *P < 0.01 versus MSCV-NC. **f** HOXB3 protein expression was measured in three primary AML blasts, which were transduced with MSCV-miR-375 or MSCV-NC. **g** HOXB3 protein expression was detected in HL-60 and THP1 cells transfected with special miR-375 inhibitor or Scramble for 48 h. (H) *HOXB3* expressions were detected in 7 leukemic cell lines, 102 primary AML blasts, and 20 NC by qRT-PCR. **i** miR-375 and *HOXB3* expressions were measured in 102 primary AML blasts and plotting of miR-375 expression versus *HOXB3* expression showed an inverse correlation between them. The correlation coefficient (R) and *P* values were detected by Pearson correlation

To determine potential miR-375-targeted genes, the microRNA.org software (http://www.microRNA. org) was utilized to predict possible target genes. HOXB3 was finally chosen for the next analysis because *HOXB3* is overexpressed in AML patients and promotes proliferation and self-renewal of mouse and human HSPCs [26, 27]. As reported, miRNAs regulate gene expression mainly through binding 3’-UTR of target genes [5]. MiR-375 potentially binds 3’-UTR of *HOXB3* (Fig. 3a), which includes putative miR-375-binding sites and was subcloned into pMIR vectors to construct wide-type vector pMIR-HOXB3’UTR and mutation vector pMIR-HOXB3’UTR (Mut). These constructs were cotransfected into 293 T cells with either miR-375 mimics or scramble. Luciferase activity was measured at 48 h after each transfection. Cells cotransfected with miR-375 mimics exhibited approximately 60% decrease of luciferase activity in comparison with those cotransfected with scramble. However, the decrease of luciferase by miR-375 was almost abolished by the mutation in miR-375-binding sites (Fig. 3b).

To explore whether miR-375 can reduce endogenous HOXB3 expression, HL-60 and THP1 cells were transduced with retrovirus vector MSCV-NC or MSCV-miR-375. To avoid supraphysiological expression of miR-375 in leukemic cell lines, we diluted the virus titer to transduce leukemic cells and selected clones with the lowest overexpression of miR-375. The expression of miR-375 is about 20–30-fold higher in leukemic cells transduced with MSCV-miR-375 than MSCV-NC (Fig. 3c). To further confirm that miR-375 is actually overexpressed in leukemic cells transduced with MSCV-miR-375, the expression of pre-miR-375 was measured. The levels of pre-miR-375 were elevated by about 30-fold in leukemic cells transduced with MSCV-miR-375 than MSCV-NC (Additional file 3: Figure S1C). Accordingly, overexpression of miR-375 decreased both the protein and mRNA expressions of HOXB3 than NC (Fig. 3d and e). Furthermore, three primary AML blasts were transduced with MSCV-miR-375 or MSCV-NC. Similarly, overexpression of miR-375 reduced the expressions of HOXB3 in all three primary AML blasts (Fig. 3f).

We then determined whether inhibition of miR-375 modulates the expression of HOXB3. Leukemic cell lines were transfected with special miR-375 inhibitor. Inhibition of miR-375 modestly increased the protein expression of HOXB3 in HL-60 and THP1 cells (Fig. 3g). Next, we asked whether HOXB3 is upregulated in AML cells. The mRNA expressions of *HOXB3* were detected in the same 7 leukemic cell lines, 102 primary AML blasts, and 20 NC. The expression of *HOXB3* was 3.0-fold higher in leukemic cell lines than in NC (*P* < 0.01, Fig. 3h) and 3.7-fold higher in primary AML blasts than in NC (*P* < 0.01, Fig. 3h). Finally, we determined whether miR-375 expression is inversely associated with *HOXB3*. When the relative expression of miR-375 was plotted against with *HOXB3*, a moderate inverse correlation between miR-375 and *HOXB3* was found in 102 patients with AML (R = − 0.249 and *P* = 0.012, Fig. 3i).

### Anti-leukemia effects of miR-375 in leukemic cells

**Fig. 4:**
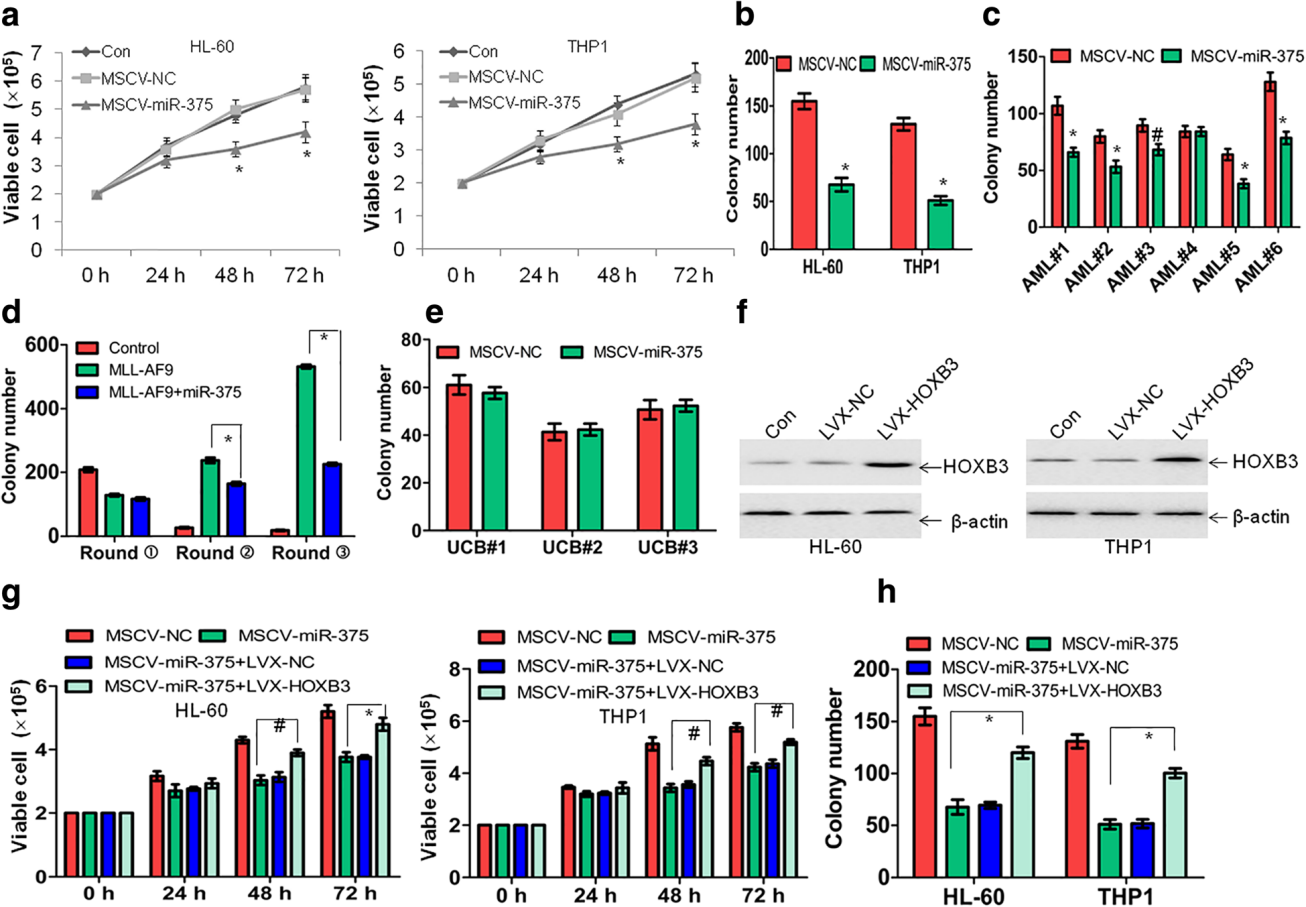
Ectopic expression of miR-375 inhibits cell growth and reduces colony formation. **a** Viable cell number was counted by the trypan-blue exclusion assay in HL-60 and THP1 cells, which were transduced with MSCV-miR-375 or MSCV-NC for the indicated times. *P < 0.01 versus MSCV-NC. **b** and **c** HL-60, THP1, and six primary AML blasts were transduced with MSCV-miR-375 or MSCV-NC, and then plated in methylcellulose for leukemia progenitor cell colony formation. *P < 0.01 and ^#^*P* < 0.05 versus MSCV-NC. **d** Normal mouse bone marrow cells were transduced with MSCV-GFP-NC, MSCV-GFP-MLL-AF9, or MSCV-GFP-MLL-AF9+ MSCV-miR-375, and colony forming/replating assays were done thereafter. *P < 0.01. **e** Colony formation was counted in CD34^+^ hematopoietic stem and progenitor cells (HSPC), which were freshly isolated from three umbilical cord blood (UCB) and transduced with MSCV-NC or MSCV-miR-375. **f** HOXB3 protein level was measured in HL-60 and THP1 cells transduced with LVX-NC or LVX-HOXB3 for 48 h. **g** The rescue experiments were performed in HL-60 and THP1 cells, which were transduced with MSCV-NC, MSCV-miR-375, MSCV-miR-375 plus LVX-NC, or MSCV-miR-375 plus LVX-HOXB3, respectively. *P < 0.01 and ^#^*P* < 0.05. **h** Colony formation was performed in HL-60 and THP1 cells following transduction with MSCV-NC, MSCV-miR-375, MSCV-miR-375 plus LVX-NC, or MSCV-miR-375 plus LVX-HOXB3, respectively. *P < 0.01

To determine whether miR-375 possesses anti-leukemia activity, proliferation and apoptosis were detected in HL-60 and THP1 cells transduced with MSCV-miR-375 or MSCV-NC. MiR-375 reduced the cell growth in leukemic cells (Fig. 4a) but did not induce cell apoptosis (data not shown). To further examine whether miR-375 affects cell cycle in leukemia cells, distribution of G0/G1, S, and G2/M phase was analyzed in HL-60 and THP1 cells transduced with MSCV-miR-375 or MSCV-NC. Overexpression of miR-375 significantly extended G0/G1 phase in HL-60 and THP1 cells (Additional file 5: Figure S2A and B), suggesting that the extended G0/G1 phase by miR-375 might inhibit proliferation of leukemia cells. Next, miR-375 notably decreased the number of colonies in leukemic cell lines (Fig. 4b) and reduced colony formation in 5 of 6 CD34^+^ leukemia stem/progenitor cells from AML patients (Fig. 4c). To further investigate the biological function of miR-375 in leukemia stem/progenitor cells, we performed a colony-forming/replating assay. Normal mouse bone marrow progenitor cells were transduced with MSCV-GFP-MLL-AF9 or MSCV-GFP-MLL-AF9 plus MSCV-miR-375 and then were plated onto methylcellulose medium, because MLL-AF9 can rapidly transform normal hematopoietic stem cells into leukemia stem cells [28]. The colonies were replated every 7 days under the same condition for another two times. Forced expression of miR-375 significantly inhibited the colony-forming capacity induced by MLL-AF9 after second and third replating (Fig. 4d). However, miR-375 did not affect the colony-forming capacity in normal CD34^+^ HSPC from umbilical cord blood (UCB, Fig. 4e). To explore whether HOXB3 plays an important role in the anti-leukemia activity by miR-375, leukemic cells were transduced with pLVX-HOXB3 or pLVX-NC. As shown in Fig. 4f, HOXB3 was significantly increased in LVX-HOXB3-transduced leukemic cells than LVX-NC-transduced cells. Furthermore, overexpression of HOXB3 partially prevented miR-375-induced cell growth arrest (Fig. 4g) and partially blocked the reduction of colony formation by miR-375 in HL-60 and THP1 cells (Fig. 4h).

### Inhibition of HOXB3-CDCA3 signaling pathway partially mimics miR-375-induced anti-leukemia activity

**Fig. 5:**
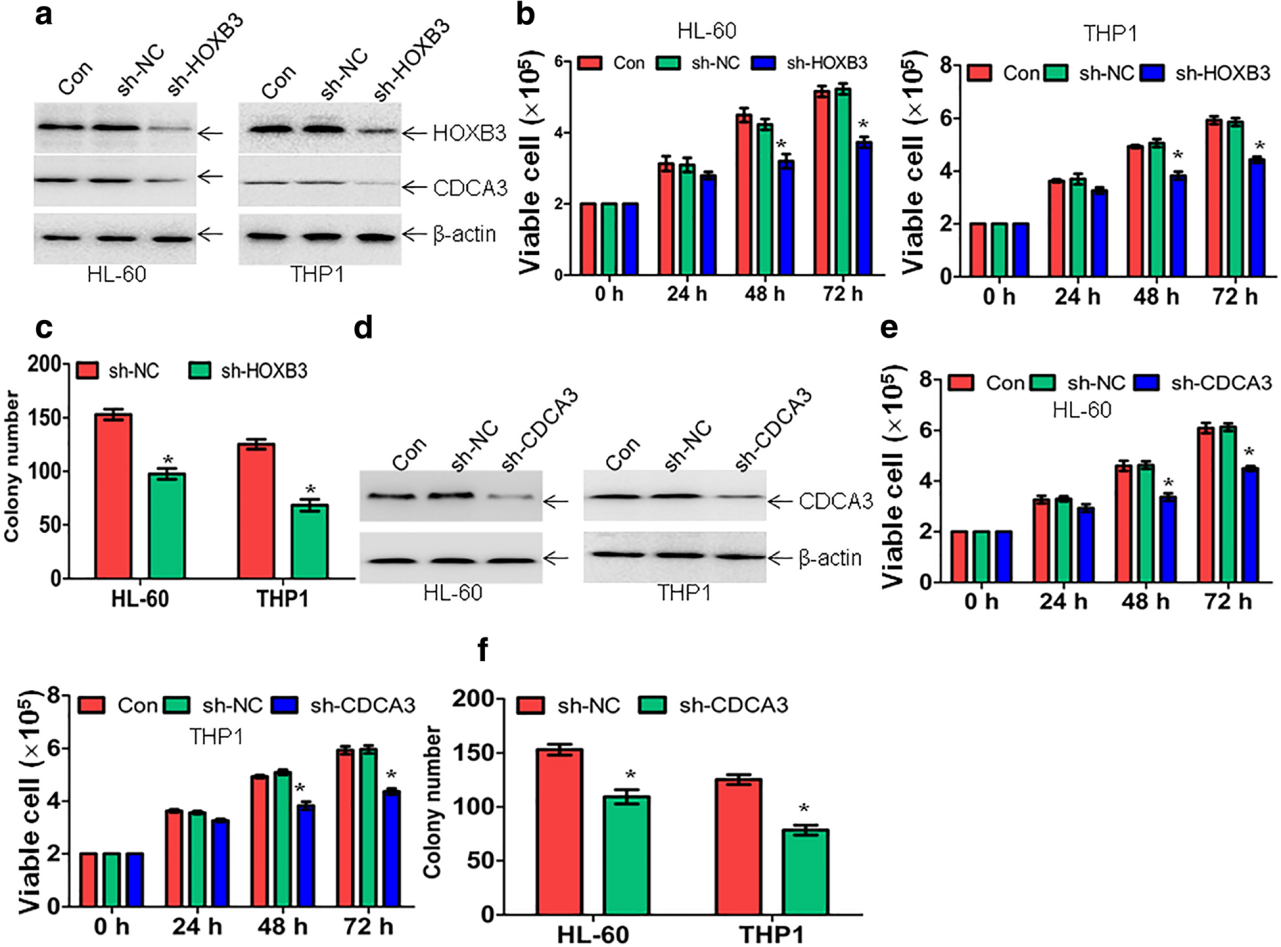
Knockdown of *HOXB3* and *CDCA3* partially mimics the anti-leukemia activity by miR-375. **a** HL-60 and THP1 cells were transduced with special shRNA for *HOXB*3 or a control shRNA (sh-NC). HOXB3 and CDCA3 expressions were detected by western blot. **b** Viable cell number by the trypan-blue exclusion assay was counted in HL-60 and THP1 cells, which were transduced with special shRNA for *HOXB3* or sh-NC for the indicated times. *P < 0.01 versus sh-NC. **c** Colony number was counted in HL-60 and THP1 cells, which were transduced with special shRNA for HOXB3 or sh-NC. *P < 0.01 versus sh-NC. **d** The protein expression of CDCA3 was detected in HL-60 and THP1 cells following transduction with special shRNA for *CDCA3* or sh-NC. **e** Viable cell number by the trypan-blue exclusion assay was counted in HL-60 and THP1 cells infected with special shRNA for *CDCA3* or sh-NC for the indicated time. *P < 0.01 versus sh-NC. **f** Colony number was counted in HL-60 and THP1 cells, which were transduced with special shRNA for *CDCA3* or sh-NC. *P < 0.01 versus sh-NC

As reported, HOXB3 facilitates cell proliferation through transcriptional activation of cell division cycle associated 3 (*CDCA3*) [17], which prevents the arrest of cell cycle progression at the G1 phase [29]. To determine whether HOXB3 regulates the expression of CDCA3 in leukemic cells, the protein expressions of HOXB3 and CDCA3 were measured in HL-60 and THP1 cells transduced with special shRNA for *HOXB3* or negative control. HOXB3 expression was significantly decreased by sh-HOXB3 followed by the downregulation of CDCA3 (Fig. 5a). Having shown that miR-375 negatively regulated HOXB3 expression and inhibited proliferation and colony formation, we then determined the role of *HOXB3* knockdown in the anti-leukemia activity. Knockdown of HOXB3 by special shRNA decreased the proliferation (Fig. 5b) and colony-formation ability (Fig. 5c) in HL-60 and THP1 cells compared with negative control. To exclude the possible unwanted off-targets by shRNA, another shRNA for *HOXB3* (sh-HOXB3#2) were constructed. As shown in Additional file 6: Figure S3A, sh-HOXB3#2 significantly reduced the expression of HOXB3 followed by the downregulation of CDCA3. Accordingly, knockdown of HOXB3 by sh-HOXB3#2 decreased the proliferation than negative control in HL-60 and THP1 cells (Additional file 6: Figure S3B). Next, we determined whether knockdown of *CDCA3* presents anti-leukemia activity. HL-60 and THP1 cells were transduced with special shRNA for *CDCA3* or negative control. The protein expression of CDCA3 was significantly decreased in sh-CDCA3-transduced cells compared with negative control (Fig. 5d). Accordingly, the decrease of CDCA3 inhibited cell growth (Fig. 5e) and reduced colony formation (Fig. 5f).

### HOXB3 contributes to the further hypermethylation of miR-375 through enhancing DNMT3B expression to bind in the pre-miR-375 promoter

**Fig. 6:**
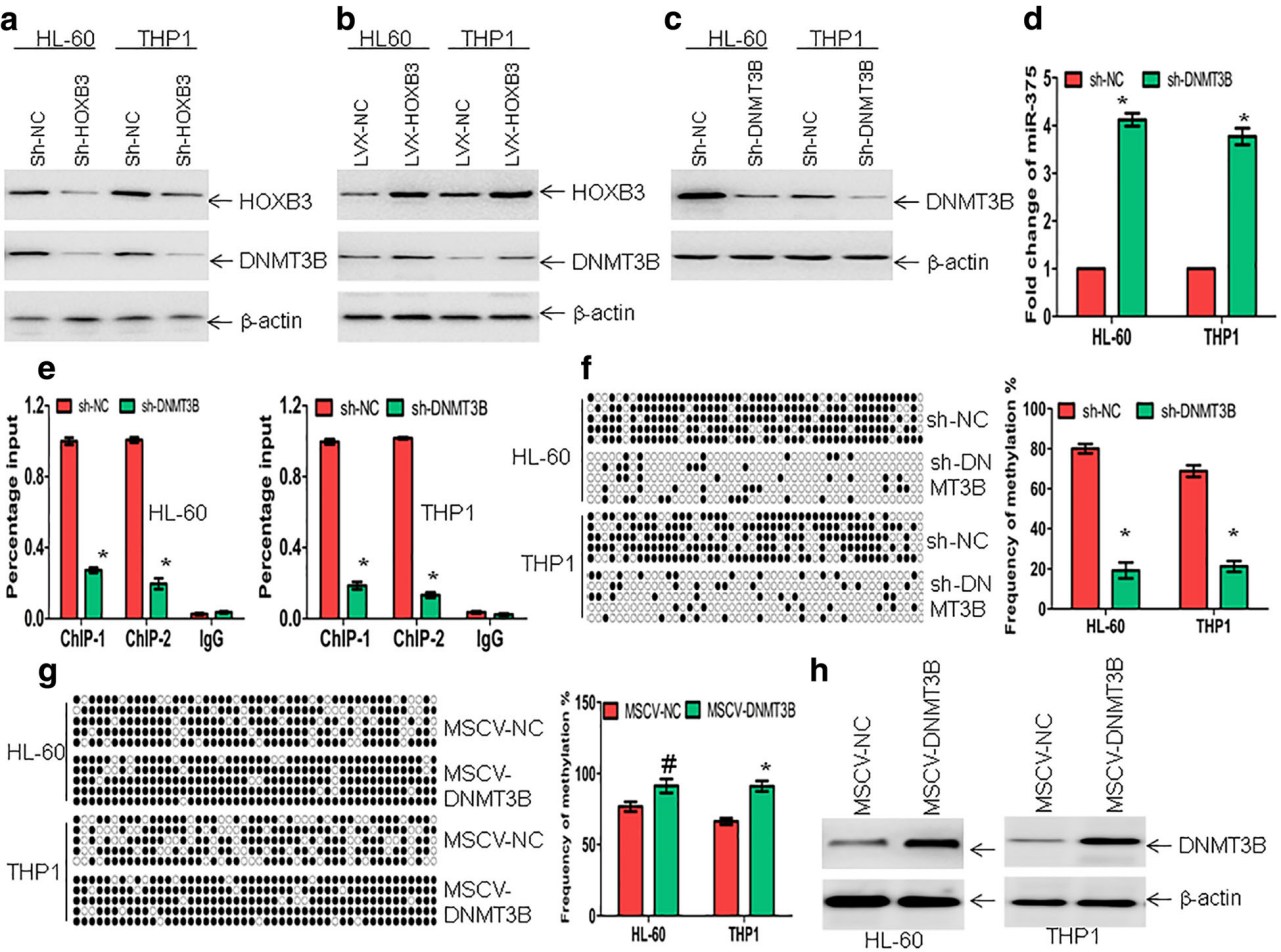
HOXB3 enhances the expression of DNMT3B to bind in pre-miR-375 promoter. **a** HL-60 and THP1 cells were transduced with special shRNA for *HOXB3* (sh-HOXB3) or sh-NC. HOXB3 and DNMT3B expressions were detected by western blot. **b** HOXB3 and DNMT3B expressions were detected in HL-60 and THP1 cells, which were transduced with overexpression vector LVX-HOXB3 or LVX-NC. **c** DNMT3B expression was detected in HL-60 and THP1 cells transduced with special shRNA targeting *DNMT3B* (sh-DNMT3B) or sh-NC. **d** MiR-375 expression was measured in HL-60 and THP1 cells transduced with sh-DNMT3B or sh-NC. *P < 0.01 versus sh-NC. **e** Soluble chromatin from HL-60 and THP1 cells, which were transduced with sh-NC or sh-DNMT3B, was immunoprecipitated with anti-DNMT3B antibody. Immunoprecipitated DNA was analyzed by qRT-PCR. *P < 0.01 versus sh-NC. **f** Bisulfite genomic sequencing was performed to detect the methylation status of the DNA sequences at -260 bp − + 136 bp in the pre-miR-375 gene upstream region in HL-60 and THP1 cells, which were transduced with sh-NC or sh-DNMT3B. Each row of circles represents the sequence of an individual clone. Black circles and empty circles represent methylated and unmethylated CpG dinucleotides, respectively (Left). Shown was the summary of frequencies of methylated CpG dinucleotides detected in HL-60 and THP1 cells by bisulfite genomic sequencing (Right). *P < 0.01 versus sh-NC. **g** Bisulfite sequencing was performed using DNA from HL-60 and THP1 cells transduced with MSCV-DNMT3B or MSCV-NC. Black circles and empty circles represent methylated and unmethylated CpG dinucleotides, respectively (Left). Shown was the summary of frequencies of methylated CpG dinucleotides detected in HL-60 and THP1cells by bisulfite genomic sequencing (Right). *P < 0.01 and ^#^*P* < 0.05 versus MSCV-NC. **h** Overexpression of DNMT3B in HL-60 and THP1 cells was indicated by western blot

HOXB3 increases the expression of DNA methyltransferase (DNMT3B), which is in turn recruited to the *RASSF1A* promoter, resulting in hypermethylation and silencing of *RASSF1A* expression in lung adenocarcinoma [18]. Therefore, we determined whether HOXB3 increases the expression of DNMT3B and further enhances DNA hypermethylation of miR-375. HL-60 and THP1 cells were transduced with special shRNA for *HOXB3* or negative control. Reduction of HOXB3 by shRNA downregulated the expression of DNMT3B (Fig. 6a). Meanwhile, the forced overexpression of HOXB3 increased the level of DNMT3B (Fig. 6b), suggesting that HOXB3 regulates the expression of DNMT3B in leukemic cells. To explore whether DNMT3B facilitates the silencing of miR-375, DNMT3B was knocked down by special shRNA. Knockdown of DNMT3B (Fig. 6c) increased the expression of miR-375 (Fig. 6d), suggesting that DNMT3B possibly binds pre-miR-375 promoter and regulates DNA hypermethylation. To exclude the possible unwanted off-targets by shRNA, another shRNA for *DNMT3B* (sh-DNMT3B#2) was constructed. Accordingly, sh-DNMT3B#2 reduced the expression of DNMT3B (Additional file 6: Figure S3C) and increased the expression of miR-375 (Additional file 6: Figure S3D). To further confirm that DNMT3B binds pre-miR-375 promoter, two pairs of primer surrounding the putative promoter of pre-miR-375 for ChIP were designed to detect the possible binding activity of DNMT3B (Additional file 7: Figure S4A). Immunoprecipitated DNA from HL-60 and THP1 cells was analyzed by qRT-PCR. Knockdown of *DNMT3B* by special shRNA substantially attenuated the binding activity of DNMT3B in pre-miR-375 promoter (Fig. 6e). To determine whether DNMT3B actually methylates these regions, bisulfite sequencing was performed in HL-60 and THP1 cells, in which DNMT3B was knocked down or overexpressed. Knockdown of DNMT3B drastically decreased the methylation frequency (Fig. 6f), while overexpression of DNMT3B increased the methylation frequency (Fig. 6g). Western blot indicated successful overexpression of DNMT3B (Fig. 6h).

Finally, we determined whether HOXB3 modulates the binding activity of DNMT3B in pre-miR-375 promoter and regulates the expression of miR-375, in turn. ChIP assay was performed using anti-DNMT3B antibody in HL-60 and THP1 cells, which were transduced with sh-NC or sh-HOXB3. Knockdown of *HOXB3* decreased the binding ability of DNMT3B in pre-miR-375 promoter (Additional file 7: Figure S4B) and subsequently increased the expression of miR-375 (Additional file 7: Figure S4C).

### Anti-leukemia effects of miR-375 in vivo

**Fig. 7:**
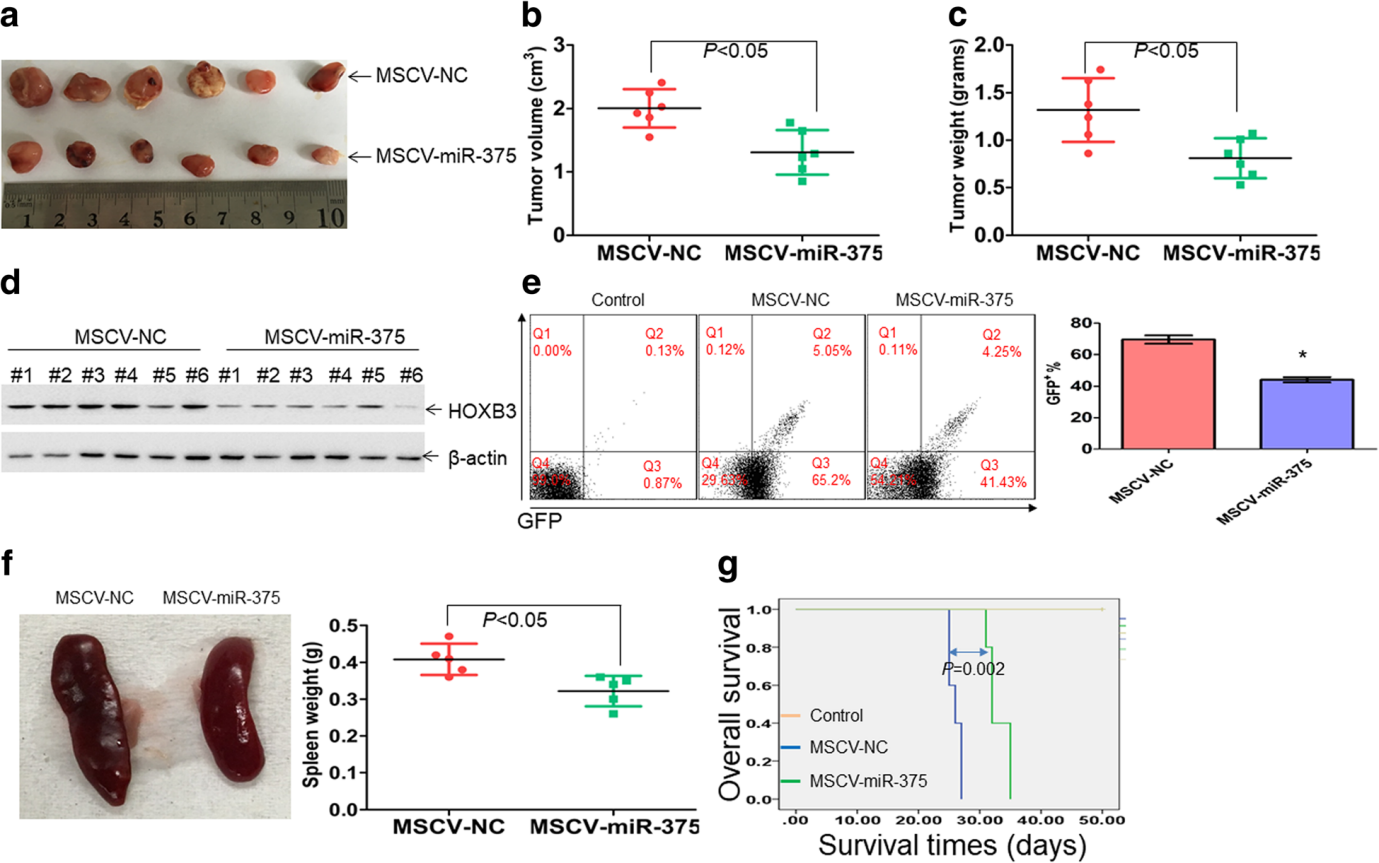
The anti-leukemia effects of miR-375 in vivo. About 1х10^7^ viable HL-60 cells transduced with MSCV-miR-375 or MSCV-NC were injected subcutaneously into right flank of each nude mouse. **a** A photograph of xenografted tumors in mice xenografted by HL-60 cells, which were transduced with MSCV-miR-375 or MSCV-NC. **b** Volumes of all xenografted tumors were measured when the experiment was terminated at 42 days after tumor cell inoculation. **c** Net weights of all xenografted tumors were measured at the termination of the experiment. **d** The protein expression of HOXB3 was detected in xenografted tumor lysates from HL-60 cells transduced with MSCV-miR-375 or MSCV-NC. **e** THP1 cells were transduced with lentivirus vector pLVX-IRES-ZsGreen1 and GFP-positive cells were sorted by flow cytometry. The GFP-positive cells were further transduced with MSCV-miR-375 or MSCV-NC and treated with puromycin for 1 week. Then, THP1-GFP-miR-375 or THP1-GFP-NC were xenografted into NSG mice. Peripheral blood cells were extracted from mice when the mice became moribund and GFP-positive cells were analyzed by flow cytometry. *P < 0.01 versus MSCV-NC. Shown is a representative plot for GFP-positive cells (Left) and summary of GFP-positive cells (Right). **f** Representative images of spleens were observed from THP1-miR-375-engrafted mice or THP1-NC-engrafted mice (Left). All the spleens in THP1-miR-375-engrafted mice or THP1-NC-engrafted mice were weighted (Right). **g** MiR-375 prolonged the overall survival time in an engrafted mice model

Finally, we determined whether forced overexpression of miR-375 inhibits the leukemogenesis in a nude mouse xenograft model. HL-60 cells were transduced with MSCV-NC or MSCV-miR-375, followed by puromycin selection for 1 week. Nude mice were divided in two groups (*N* = 6 per group) and inoculated with HL-60-miR-375 or HL-60-NC. Tumors in mice inoculated with HL-60-miR-375 were considerably smaller than those in mice inoculated with negative control (Fig. 7a). Furthermore, tumor growth was suppressed in mice inoculated with HL-60-miR-375 than control mice (Additional file 8: Figure S5A). Similarly, the average tumor volume was reduced by 34.7% (Fig. 7b) and average tumor weight was reduced by 38.5% (Fig. 7c) in HL-60-miR-375-inoculated mice compared with control mice. Consistent with the results from cell lines, the protein levels of HOXB3 were considerably decreased in mice inoculated with HL-60-miR-375 compared with control mice (Fig. 7d).

To further examine the effects of miR-375 overexpression in vivo, THP1 cells were transduced with a lentivirus encoding enhanced GFP followed by flow cytometric sorting for GFP-positive cells, which were further transduced with MSCV-NC or MSCV-miR-375. THP1-miR-375 or THP1-NC cells were xenografted into nonobese diabetic scid gamma (NSG) mice. We detected GFP cells in peripheral blood when mice transplanted with THP1-NC became moribund. The percentage of GFP-positive cells was reduced by 35% in mice transplanted with THP1-miR-375 than in mice transplanted with THP1-NC (Fig. 7e). We then evaluated whether miR-375 reduced the infiltration of blast cells in the spleen. The spleen volume and weight were reduced in THP1-miR-375-transplanted mice than in THP1-NC-transplanted mice (Fig. 7f). Extensive infiltrations of immature blast cells destroyed the normal architectures in spleens from THP1-NC-transplanted mice (Additional file 8: Figure S5B, Left). Accordingly, forced expression of miR-375 substantially decreased the infiltrations (Additional file 8: Figure S5B, Right). Finally, miR-375 significantly extended the survival time in mice (*P* < 0.01; Fig. 7g).

## Discussion

**Fig. 8:**
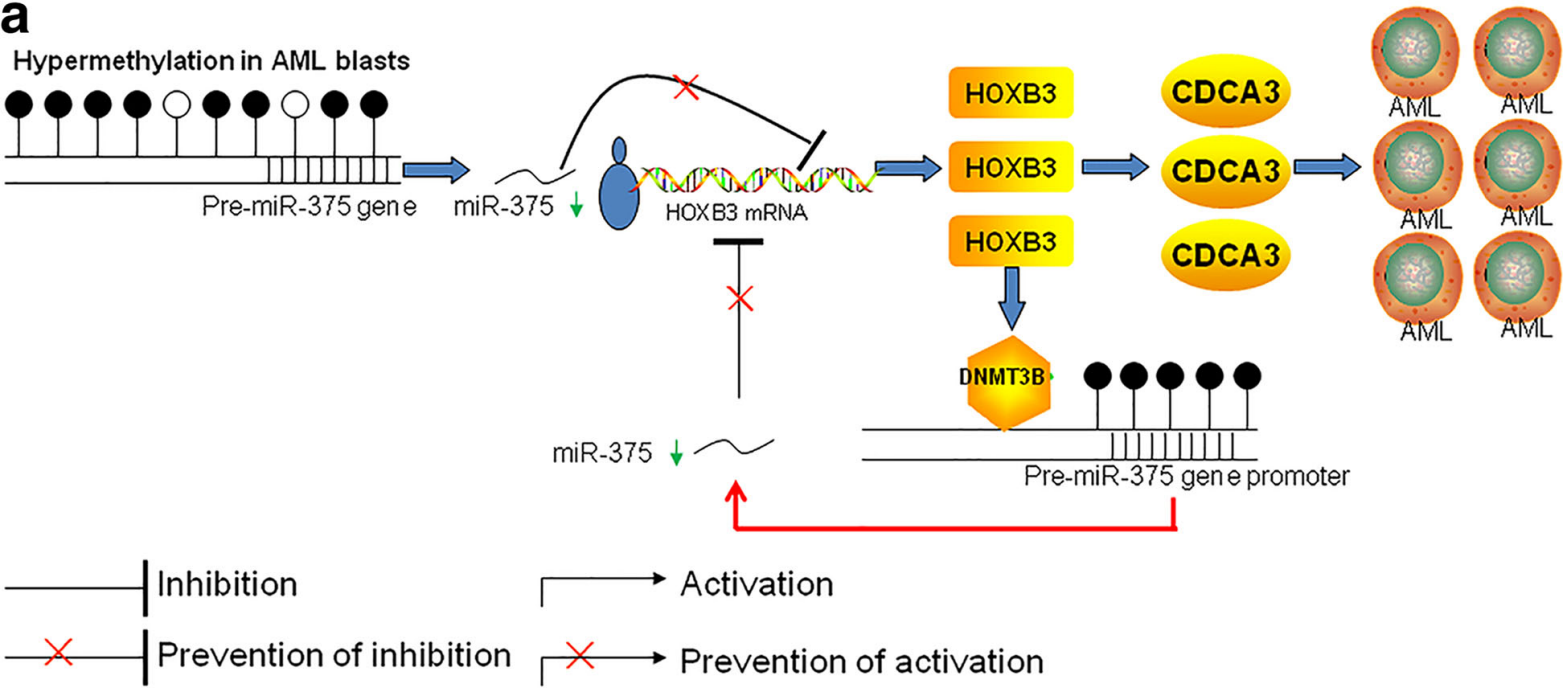
An illustration of miR-375-HOXB3-CDCA3/DNMT3B regulatory circuitry. **a** The decreased expression of miR-375 due to DNA hypermethylation results in the high expression of HOXB3, contributing to cell proliferation and colony formation through increasing the expression of CDCA3. Moreover, HOXB3 enhances and recruits DNMT3B to bind in pre-miR-375 promoter, leading to further DNA hypermethylation and subsequent downregulation of miR-375 in AML cells, in turn

In the present study, we have identified a novel miR-375-HOXB3-CDCA3/DNMT3B regulatory circuitry in AML cells. Dysregulation of this regulatory circuitry facilitates leukemogenesis. DNA hypermethylation of the pre-miR-375 promoter results in the low expression of miR-375, which leads to the inability to decrease HOXB3 expression and finally causes high expression of HOXB3. High level of HOXB3 enhances CDCA3 expression to facilitate cell proliferation and colony formation. Furthermore, high level of HOXB3 enhances DNMT3B expression to bind in pre-miR-375 promoter, leading to the further DNA hypermethylation and lower expression of miR-375, in turn (Fig. 8a). In normal control cells or AZA-treated leukemic cells, DNA hypomethylation in the pre-miR-375 promoter restores the expression of miR-375, resulting in the downregulation of HOXB3. Low expression of HOXB3 reduces the CDCA3 expression to inhibit cell growth and colony formation. Moreover, low expression of HOXB3 decreases DNMT3B expression and weakens the binding activity of DNMT3B in the pre-miR-375 promoter, resulting in the further DNA hypomethylation and subsequent higher expression of miR-375, in turn (Additional file 9: Figure S6A). Thus, restoring the expression of miR-375 has anti-leukemia ability by disrupting the miR-375-HOXB3-CDCA3/DNMT3B regulatory circuitry.

miR-375 possesses significant anti-leukemia or anti-tumor activity through modulating multiple target genes, including *JAK2* [10], *SP1* [30], *PIK3CA* [31], and *AEG-1* [32]. For example, forced expression of miR-375 suppresses cell proliferation, blocks G1-to-S cell cycle transition, and inhibits cell migration and invasion via targeting transcription factor *SP1* [30]. Overexpression of miR-375 in liver cancer cells decreases cell proliferation, clonogenicity, and migration/invasion through inhibiting the expression of *astrocyte elevated gene-1* (*AEG-1*) [32]. Consistent with previous results that miR-375 presents anti-cancer activity through targeting *HOXB3* in pancreatic cancer and breast cancer [33, 34], our data indicate that overexpression of miR-375 inhibits cell proliferation and colony formation through inhibiting HOXB3 expression. Overexpression of HOXB3 partially prevents anti-leukemia effects by miR-375 and knockdown of HOXB3 resembles the anti-leukemia activities of miR-375, suggesting that HOXB3 is functionally important target of miR-375. However, whether miR-375 inhibits leukemia stem cell is not determined. MiR-375 reduces the colony number in leukemic cell lines and primary AML blasts. In addition, miR-375 decreases the colony formation induced by MLL-AF9 fusion oncogene in the replating assay, suggesting that miR-375 might inhibit self-renew of leukemia stem cells via decreasing the colony formation. Importantly, miR-375 can not decrease colony formation in normal CD34^+^ HSPC cells, suggesting that restoring miR-375 expression inhibits self-renew of leukemia stem cell but not of normal CD34^+^ cells. Thus, eradicating leukemia stem cell by overexpression of miR-375 combined with chemotherapeutics might facilitate the successful treatment because AML originates from leukemia stem cell [35]. Although most studies indicate that miR-375 acts as a tumor suppressor, several other studies suggest that miR-375 is an oncogene and facilitates cancer initiation and progression [36, 37]. For example, overexpression of miR-375 promotes cell migration and invasion in lung cancer cells [36]. These discrepancies suggest the important role of cellular context on the function of miRNAs.

Dysregulation of miRNA is frequently observed in various types of cancer. In many human cancers, miR-375 is aberrantly downregulated due to epigenetic modulation including DNA hypermethylation and histone deacetylation. DNA hypermethylation of pre-miR-375 in melanoma patient tissue samples causes the low expression of miR-375 [38]. Our published data indicate that histone deacetylation (HDAC) inhibitor restores miR-375 expression, suggesting that histone acetylation might modulate the expression of miR-375 [13]. In addition, miR-375 expression was also modulated by some transcription factors [39, 40]. These data suggest that miR-375 may widely mediate the different biological progress. DNA hypermethylation of pre-miR-375 promoter occurs in leukemic cell lines and primary AML blasts but not in normal control cells. Furthermore, DNA methyltransferase inhibitor AZA restores the expression of miR-375, suggesting that hypermethylation leads to the low expression of miR-375 in leukemic cells. AZA causes the DNA hypomethylation through inhibiting the activity of DNMTs, including DNMT1, DNMT3A, and DNMT3B. Knockdown of DNMT3B increases the expression of miR-375, confirming that DNA hypermethylation mediates the silencing of miR-375. Thus, restoration of miR-375 by DNA methyltransferase inhibitors might contribute to the treatment of AML.

In addition to having anti-tumor activity, different expression of miR-375 can also predict clinical outcome in different tumors. For example, lower expression of miR-375 is associated with adverse outcome in hepatocellular carcinoma patients [41] and in non-small cell lung cancer [42]. However, little data show its role of predicting outcome in AML patients. Our data indicate that lower expression of miR-375 predicts poor clinical outcome in AML patients, suggesting that miR-375 is a novel prognostic indicator in AML. The combination of miR-375 and other genes will further stratify AML samples for a better clinical prognostication. Most studies indicate that miR-375 acts as tumor suppressor gene and lower expression of miR-375 predicts adverse outcome. However, Wang et al. reported that higher expression of miR-375 is associated with shorter OS and DFS in pediatric AML patients [43]. The possible reason for this discrepancy might be attributed to the different approaches used for profiling gene expression, the heterogeneity of patient cohorts, or the cut-off values used to define the miR-375 expression level. Further studies are thus needed to resolve this discrepancy.

The critical role of miRNAs in cancer and their involvement in common cellular pathways make them valuable and comprehensive targets. Single miRNA can target multiple genes. On the other hand, a single mRNA molecule can be modulated by multiple miRNAs. In addition to miR-375, miR-7 and miR-218 target *HOXB3* in breast cancer cells [44] and miR-10b binds 3′-UTR of *HOXB3* and decrease HOXB3 expression in endometrial cancer [45]. Finding all the miRNAs targeting *HOXB3* and understanding the role of miRNAs-HOXB3 interaction in AML cells will provide new insights in the pathogenesis and therapy of AML.

As a transcript factor, *HOXB3* can either activate or repress multiple target genes including *CDCA3* [17], *DNMT3B* [18], and *Otx2* [46], depending on the sequence of the binding site, the partner proteins, and cellular context. In AML, expression of HOXB3 is increased in most progenitor cells [47]. Consistent with a previous report [17], our data show that HOXB3 positively regulates the expression of CDCA3 and knockdown of *CDCA3* reduces cell proliferation and colony formation. Therefore, we speculate that HOXB3 enhances cell proliferation and colony formation through activating CDCA3, which prevents the arrest of cell-cycle progression at the G0/G1 phase through decreasing the expressions of the cyclin-dependent kinase inhibitors [29]. Meanwhile, HOXB3 enhances the expression of DNMT3B to promote DNA hypermethylation of tumor suppressor gene [18]. Therefore, HOXB3-CDCA3/DNMT3B signaling pathway might play an important role in the proliferation and colony formation of leukemia stem and progenitor cells.

## Conclusions

Collectively, our results delineate a complex signaling network about miR-375-HOXB3-CDCA3/ DNMT3B regulatory circuitry, in which miR-375 contributes as essential gene expression modulator. HOXB3-CDCA3/DNMT3B signaling pathway contributes to leukemogenesis and maintenance of leukemia. Thus, inhibition of HOXB3-CDCA3/DNMT3B signaling pathway might improve treatment outcomes in AML patients through restoring the expression of miR-375, such as by clinically applicable nanoparticles packaged with miR-375 mimic oligo.

## Additional files

Additional file 1: **Table S1.** AML patients’ characteristics. (DOCX 20 kb)

Additional file 2: **Table S2.** The sequences of primers. (DOCX 25 kb)

Additional file 3: **Figure S1.** The expression of miR-126 and miR-375 in normal controls, CpG islands around the region encoding miR-375, and expression of pre-miR-375. (A) The expressions of miR-126 and miR-375 were detected in all CD34^+^ cells from 20 normal controls (NC). Housekeeping gene U6 is used as a reference. The lowest expression of miR-375 in one NC was set to 1.0 and then the expressions of miR-375 and miR-126 in all other specimens were normalized by this lowest specimen. The fold change of miR-375 and miR-126 were calculated by Student’s t-test. (B) CpG islands around the region encoding pre-miR-375 were analyzed by MethPrimer software. (C) The expression of pre-miR-375 was detected by qRT-PCR in HL-60 and THP1 cells, which were transduced with MSCV-miR-375 or MSCV-NC. *P < 0.01 versus MSCV-NC. (TIFF 221 kb)

Additional file 4: **Table S3.** Multivariate analyses of overall survival (OS) and disease-free survival (DFS) of AML. (DOCX 19 kb)

Additional file 5: **Figure S2.** Cell cycle in HL-60 and THP1 cells transduced with MSCV-NC or MSCV-miR-375. (A and B) Distribution of cells were recorded in different stage of cell cycle analyzed using flow cytometry in HL-60 and THP1 cells, which were transduced with MSCV-NC or MSCV-miR-375. Shown is a representative cell cycle (A) and summary of G0/G1, S, and G2/M phases (B). *P < 0.05 verse MSCV-NC. (TIFF 304 kb)

Additional file 6: **Figure S3.** Knockdown of HOXB3 and DNMT3B by another shRNA. (A) HL-60 and THP1 cells were transduced with another special shRNA for *HOXB*3 (sh-HOXB3#2) or a control shRNA (sh-NC). HOXB3 and CDCA3 expressions were detected by western blot. (B) Viable cell number by the trypan-blue exclusion assay was counted in HL-60 and THP1 cells, which were transduced with sh-HOXB3#2 or sh-NC for the indicated times. *P < 0.01 versus sh-NC. (C) HL-60 and THP1 cells were transduced with another special shRNA for *DNMT3B* (sh-DNMT3B#2) or a control shRNA (sh-NC). DNMT3B expression was detected by western blot. (D) MiR-375 expression was measured in HL-60 and THP1 cells transduced with sh-DNMT3B#2 or sh-NC. *P < 0.01 versus sh-NC. (TIFF 345 kb)

Additional file 7: **Figure S4.** HOXB3 enhances the expression of DNMT3B to bind in pre-miR-375 promoter. (A) A schematic representation of the promoter regions amplified by ChIP-PCR assay. (B) HL-60 and THP1 cells were transduced with sh-NC or sh-HOXB3. Soluble chromatin from these cells was immunoprecipitated with anti-DNMT3B antibody. Immunoprecipitated DNA was analyzed by qRT-PCR. *P < 0.01 versus sh-NC. (C) The expression of miR-375 was detected in HL-60 and THP1 cells transduced with sh-NC or sh-HOXB3. *P < 0.01 versus sh-NC. (TIFF 310 kb)

Additional file 8: **Figure S5.** The anti-leukemia effects of miR-375 in vivo. (A) Volumes of all tumors were detected every 3 days after 2 weeks. *P < 0.05 versus MSCV-NC. (B) HE staining was taken to indicate the infiltration of THP1 leukemic cells in murine spleens. (TIFF 367 kb)

Additional file 9: **Figure S6.** An illustration of miR-375-HOXB3-CDCA3/DNMT3B regulatory circuitry. (A) In normal hematological cells or leukemic cells treated with AZA, miR-375 expression is increased because of DNA hypomethylation. Upregulation of miR-375 inhibits HOXB3 expression, resulting in the arrest of proliferation and reduction of colony formation via decreasing the expression of CDCA3. Moreover, the decreased expression of HOXB3 can not increase and recruit DNMT3B to bind in the pre-miR-375 promoter and maintains the hypomethylation of pre-miR-375, which finally leads to the upregulated expression of miR-375, in turn. (TIFF 592 kb)

## Abbreviations

3’-UTR: 3′-untranslated regions
AML: Acute myeloid leukemia
CDCA3: cell division cycle associated 3
ChIP: Chromatin immunoprecipitation
CR: complete remission
DFS: disease-free survival
DNMT3B: DNA methyltransferase 3B
HOX: homeobox
HSPC: hematopoietic stem and progenitor cells
miRNAs: microRNAs
MPN: myeloproliferative neoplasm
NSG: NOD/SCID-IL2Rγ
OS: Overall survival
pre-miR-375: precursor-miR-375
qRT-PCR: Quantitative reverse transcriptase PCR
UCB: umbilical cord blood
