# Supplementary material for: A novel miR-375-HOXB3-CDCA3/DNMT3B regulatory circuitry contributes to leukemogenesis in acute myeloid leukemia
Source: BMC Cancer. 2018 Feb 13;18:182. doi: 10.1186/s12885-018-4097-z (PMC5811974; doi:10.1186/s12885-018-4097-z)
Supplement: Supplementary file 1 — Table S1. AML patients’ characteristics. (DOCX 20 kb) [file 12885_2018_4097_MOESM1_ESM.docx]

**Table S1.** AML patients’ characteristics

| Characteristics | All patients N (%) |
| --- | --- |
| Overall | 102 |
| Gender |  |
| Male | 47 (46.1) |
| Female | 55 (53.9) |
| Age (range) | 46 (21-60) |
| FAB |  |
| M0 | 0 (0.0) |
| M1 | 12 (11.8) |
| M2 | 28 (27.5) |
| M3 | 9 (8.8) |
| M4 | 29 (28.4) |
| M5 | 22 (21.6) |
| Biphenotipic | 0 (0.0) |
| Unclassified | 2 (2.0) |
| Cytogenetics |  |
| Normal karyotype | 48 (47.1) |
| t(15;17) | 9 (8.8) |
| t(8;21) | 13 (12.7) |
| Inv(16) | 5 (4.9) |
| +8 | 6 (5.9) |
| +11 | 3 (2.9) |
| +21 | 3 (2.9) |
| Complex karyotype | 14 (13.7) |
| Not available | 2 (2.0) |
| Molecular genetic abnormality |  |
| PML-RARα | 9 (8.8) |
| AML1-ETO | 13 (12.7) |
| CBFB-MYH11 | 5 (4.9) |
| WT1 mutation | 9 (8.8) |
| FLT3-ITD mutation | 8 (7.8) |
| FLT3-TKD mutation | 2 (2.0) |
| CEBPA mutation | 19 (18.6) |
| TET2 mutation | 15 (14.1) |
| MLL-PTD mutation | 5 (4.9) |
| c-kit mutation | 7 (6.9) |
| NPM1 mutation | 8(7.8) |
