# Supplementary material for: A novel miR-375-HOXB3-CDCA3/DNMT3B regulatory circuitry contributes to leukemogenesis in acute myeloid leukemia
Source: BMC Cancer. 2018 Feb 13;18:182. doi: 10.1186/s12885-018-4097-z (PMC5811974; doi:10.1186/s12885-018-4097-z)
Supplement: Supplementary file 2 — Table S2. The sequences of primers. (DOCX 25 kb) [file 12885_2018_4097_MOESM2_ESM.docx]

| Primer | Sequence (5'-3') |
| --- | --- |
| HOXB3 3′UTR-L | GGA CTA GTC AAA GGA CAT TGT GTT TCC TGT C |
| HOXB3 3′UTR-R | CCC AAG CTT ACA GTG ACG ATT AGG AGC TGA |
| MSCV-miR-375-L | GAA GAT CTT CGA CGT GTC AGC CGC AGA T |
| MSCV-miR-375-R | GGA ATT CCC CCG TAT TAC GAC GCA GAA T |
| MSCV-DNMT3B-L | GAA GAT CTA TGA AGG GAG ACA CCA GGC A |
| MSCV-DNMT3B-R | CGG AAT TCC TAT TCA CAT GCA AAG TAG T |
| LVX-HOXB3-L | CGG AAT TCA TGC AGA AAG CCA CCTA CTA |
| LVX-HOXB3-R | GCT CTA GAT CAC AGG TGT GTT AAT TTG |
| sh-HOXB3 | GAA TCC AAG AAG CGC CCA AAT |
| sh-HOXB3#2 | CAC CCT CAC CAA ACA GAT ATT |
| sh-CDCA3 | GAA ACA GCT GAG TGA AGT ATT |
| sh-DNMT3B | GAC GAT GGC TAT CAG TCT TAC |
| sh-DNMT3B#2 | ACA CGC AAC CAG TGG TTA ATA |
| HOXB3-L | ATG CAG GGC AGT CCG GTG TA |
| HOXB3-R | GGT GAT GGG AAA GGT GGT TG |
| ChIP-1L | TTC TGT CCT CTG CTT CTC GG |
| ChIP-1R | TGT CAC TTT TCC ACC CAC CT |
| ChIP-2L | AGG TGT GAG TGT GTG TGT CT |
| ChIP-2R | GGG CTC CGT GTG CTC TTA TA |
| miR-375MMSP-L^#^ | ATT GTA GTT GGA TTG AGA TTT GGT C |
| miR-375MMSP-R^#^ | TAC CCA ATA AAC ACC TAC TTC GC |
| miR-375UMSP-L^#^ | ATT GTA GTT GGA TTG AGA TTT GGT T |
| miR-375UMSP-R^#^ | CCT ACC CAA TAA ACA CCT ACT TCA C |
| miR-375-sequence-L^$^ | CGG GCG GAG GGG TTA GGA AA |
| miR-375-sequence-R^$^ | AAC GCA CAA CCT CTC CCA CC |

**Supplemental Table 2: The sequences of primers**

^#^ for methylation-specific PCR analysis

^$^ for bisulfite-sequencing PCR analysis
