# Supplementary figures and images for: A novel miR-375-HOXB3-CDCA3/DNMT3B regulatory circuitry contributes to leukemogenesis in acute myeloid leukemia
Source: BMC Cancer. 2018 Feb 13;18:182. doi: 10.1186/s12885-018-4097-z (PMC5811974; doi:10.1186/s12885-018-4097-z)

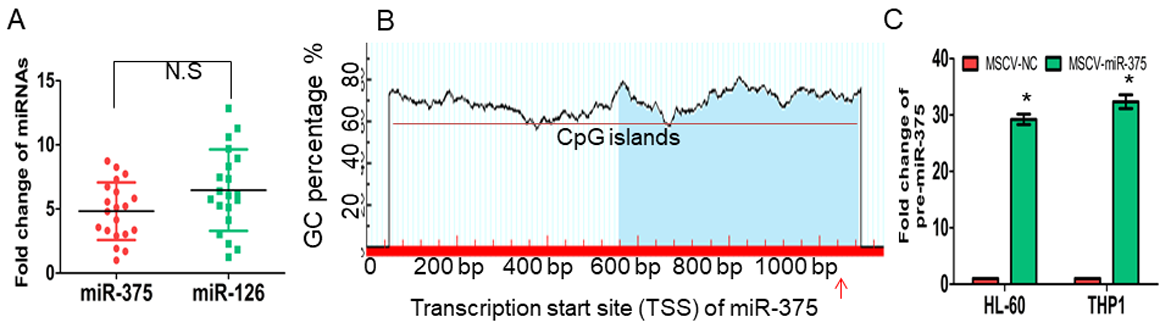

Supplement: Supplementary file 3 — Figure S1. The expression of miR-126 and miR-375 in normal controls, CpG islands around the region encoding miR-375, and expression of pre-miR-375. (A) The expressions of miR-126 and miR-375 were detected in all CD34+ cells from 20 normal controls (NC). Housekeeping gene U6 is used as a reference. The lowest expression of miR-375 in one NC was set to 1.0 and then the expressions of miR-375 and miR-126 in all other specimens were normalized by this lowest specimen. The fold change of miR-375 and miR-126 were calculated by Student’s t-test. (B) CpG islands around the region encoding pre-miR-375 were analyzed by MethPrimer software. (C) The expression of pre-miR-375 was detected by qRT-PCR in HL-60 and THP1 cells, which were transduced with MSCV-miR-375 or MSCV-NC. *P < 0.01 versus MSCV-NC. (TIFF 221 kb) [file 12885_2018_4097_MOESM3_ESM.tif]

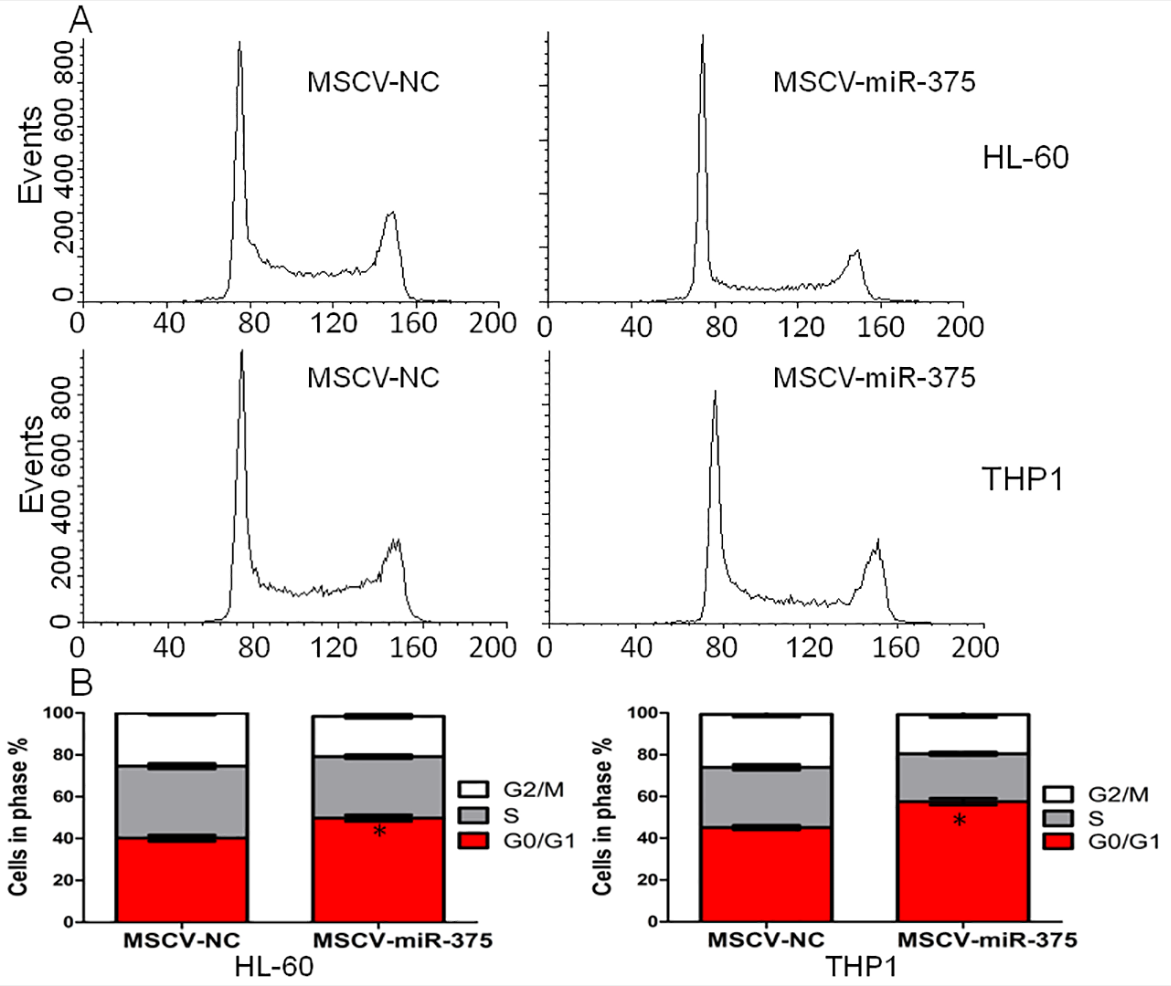

Supplement: Supplementary file 5 — Figure S2. Cell cycle in HL-60 and THP1 cells transduced with MSCV-NC or MSCV-miR-375. (A and B) Distribution of cells were recorded in different stage of cell cycle analyzed using flow cytometry in HL-60 and THP1 cells, which were transduced with MSCV-NC or MSCV-miR-375. Shown is a representative cell cycle (A) and summary of G0/G1, S, and G2/M phases (B). *P < 0.05 verse MSCV-NC. (TIFF 304 kb) [file 12885_2018_4097_MOESM5_ESM.tif]

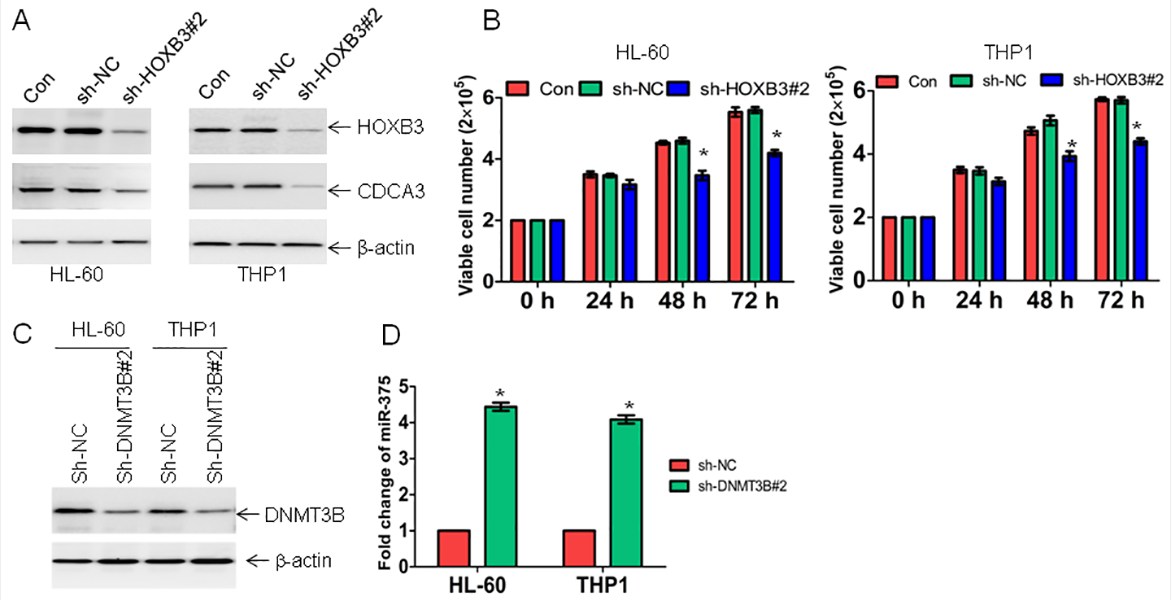

Supplement: Supplementary file 6 — Figure S3. Knockdown of HOXB3 and DNMT3B by another shRNA. (A) HL-60 and THP1 cells were transduced with another special shRNA for HOXB3 (sh-HOXB3#2) or a control shRNA (sh-NC). HOXB3 and CDCA3 expressions were detected by western blot. (B) Viable cell number by the trypan-blue exclusion assay was counted in HL-60 and THP1 cells, which were transduced with sh-HOXB3#2 or sh-NC for the indicated times. *P < 0.01 versus sh-NC. (C) HL-60 and THP1 cells were transduced with another special shRNA for DNMT3B (sh-DNMT3B#2) or a control shRNA (sh-NC). DNMT3B expression was detected by western blot. (D) MiR-375 expression was measured in HL-60 and THP1 cells transduced with sh-DNMT3B#2 or sh-NC. *P < 0.01 versus sh-NC. (TIFF 345 kb) [file 12885_2018_4097_MOESM6_ESM.tif]

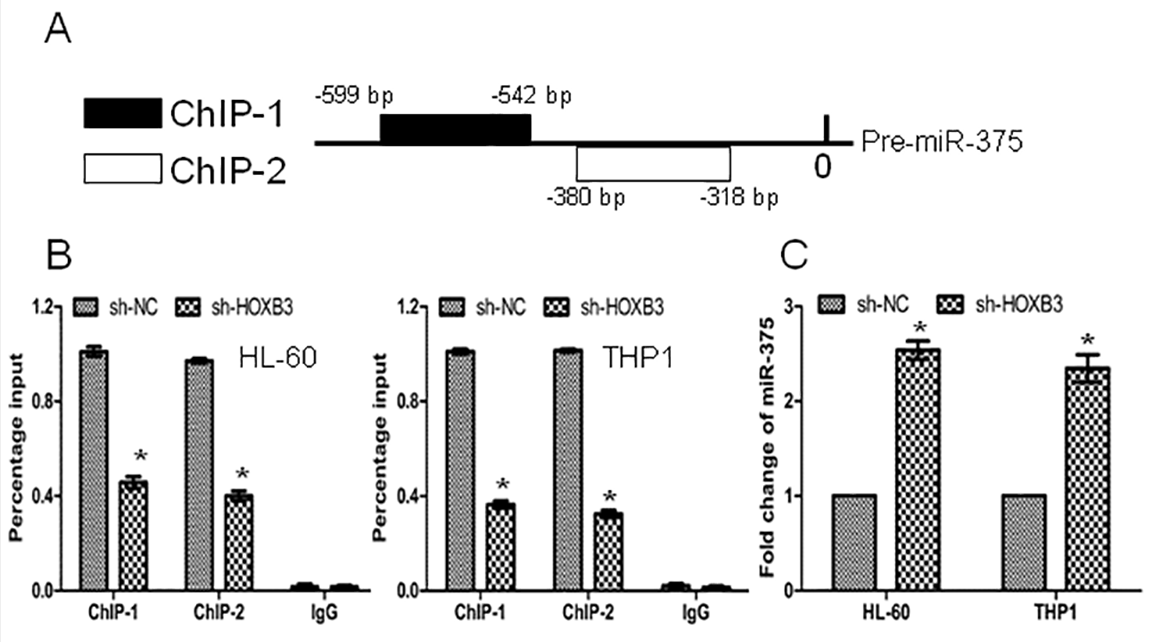

Supplement: Supplementary file 7 — Figure S4. HOXB3 enhances the expression of DNMT3B to bind in pre-miR-375 promoter. (A) A schematic representation of the promoter regions amplified by ChIP-PCR assay. (B) HL-60 and THP1 cells were transduced with sh-NC or sh-HOXB3. Soluble chromatin from these cells was immunoprecipitated with anti-DNMT3B antibody. Immunoprecipitated DNA was analyzed by qRT-PCR. *P < 0.01 versus sh-NC. (C) The expression of miR-375 was detected in HL-60 and THP1 cells transduced with sh-NC or sh-HOXB3. *P < 0.01 versus sh-NC. (TIFF 310 kb) [file 12885_2018_4097_MOESM7_ESM.tif]

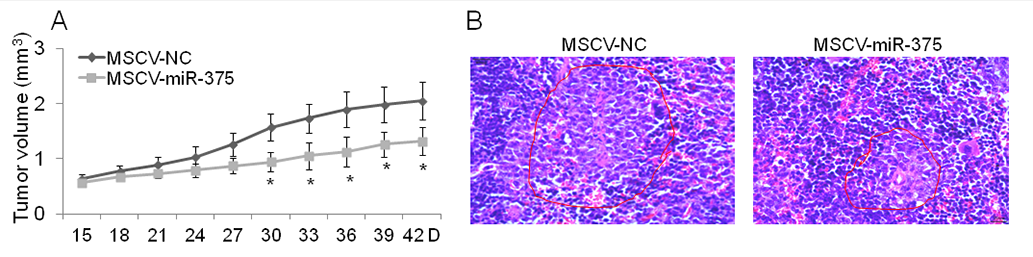

Supplement: Supplementary file 8 — Figure S5. The anti-leukemia effects of miR-375 in vivo. (A) Volumes of all tumors were detected every 3 days after 2 weeks. *P < 0.05 versus MSCV-NC. (B) HE staining was taken to indicate the infiltration of THP1 leukemic cells in murine spleens. (TIFF 367 kb) [file 12885_2018_4097_MOESM8_ESM.tif]

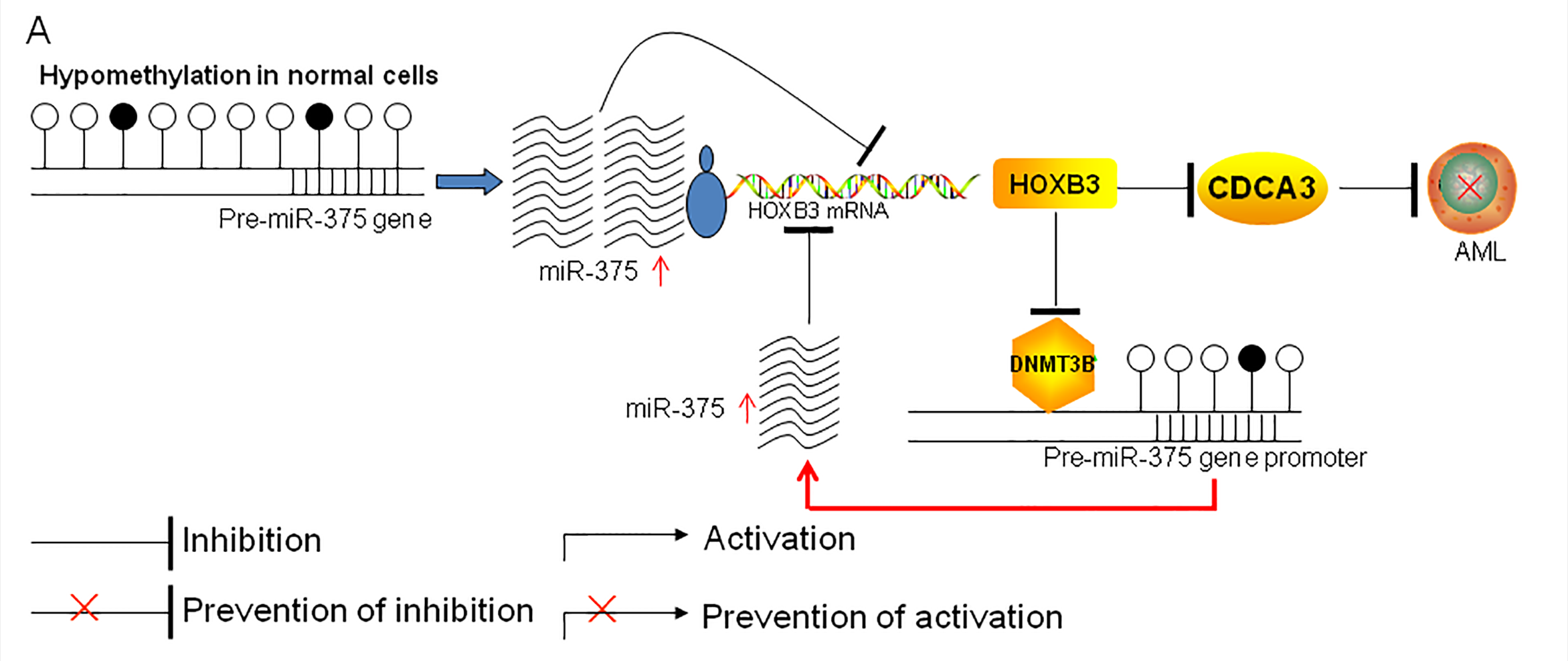

Supplement: Supplementary file 9 — Figure S6. An illustration of miR-375-HOXB3-CDCA3/DNMT3B regulatory circuitry. (A) In normal hematological cells or leukemic cells treated with AZA, miR-375 expression is increased because of DNA hypomethylation. Upregulation of miR-375 inhibits HOXB3 expression, resulting in the arrest of proliferation and reduction of colony formation via decreasing the expression of CDCA3. Moreover, the decreased expression of HOXB3 can not increase and recruit DNMT3B to bind in the pre-miR-375 promoter and maintains the hypomethylation of pre-miR-375, which finally leads to the upregulated expression of miR-375, in turn. (TIFF 592 kb) [file 12885_2018_4097_MOESM9_ESM.tif]
