# Supplementary material for: A novel miR-375-HOXB3-CDCA3/DNMT3B regulatory circuitry contributes to leukemogenesis in acute myeloid leukemia
Source: BMC Cancer. 2018 Feb 13;18:182. doi: 10.1186/s12885-018-4097-z (PMC5811974; doi:10.1186/s12885-018-4097-z)
Supplement: Supplementary file 4 — Table S3. Multivariate analyses of overall survival (OS) and disease-free survival (DFS) of AML. (DOCX 19 kb) [file 12885_2018_4097_MOESM4_ESM.docx]

Table S3: Multivariate analyses of overall survival (OS) and disease-free survival (DFS) of AML patients

| Analysis | OS  HR 95% CI *P* | DFS  HR 95% CI *P* |
| --- | --- | --- |
| MiR-375 (low versus high) | 2.58 1.05–4.87 <0.01 | 2.13 1.05–4.14 <0.05 |
| Sex^*^ | 0.33 0.17-0.94 >0.05 | 0.43 0.21-1.15 >0.05 |
| Age^#^ | 0.39 0.11-0.79 >0.05 | 0.65 0.37-1.05 >0.05 |
| karyotype^$^ | 0.51 0.32-0.88 >0.05 | 0.41 0.23-0.71 >0.05 |
| WT1 mutation^**^ | 1.34 0.75-1.56 <0.05 | 1.16 0.54-1.34 <0.05 |
| FLT3 mutation^&^ | 0.45 0.31-0.94 >0.05 | 0.54 0.48-1.37 >0.05 |
| CEBPA mutation^##^ | 2.60 1.56-4.33 >0.05 | 1.87 1.16-3.68 >0.05 |
| NPM^***^ | 0.76 0.45-0.98 >0.05 | 0.85 0.64-1.27 >0.05 |

^*^Male versus female

^#^Age more than 46 years relative to age less than or equal to 46 years

^$^Normal karyotype versus abnormal karyotype.

^**^WT1-positve mutation versus negative mutation

^&^FLT3-positive mutation versus negative mutation

^##^CEBPA-positive mutation versus negative mutation

^***^NPM-positive mutation versus negative mutation

HR, Hazard ratio; 95% CI, 95% confidence interval
